# Anti-Diabetic Indole-Terpenoids From *Penicillium* sp. HFF16 Isolated From the Rhizosphere Soil of *Cynanchum bungei* Decne

**DOI:** 10.3389/fchem.2021.792810

**Published:** 2022-02-08

**Authors:** Na Xiao, Yiru Xu, Xinru Zhang, Haonan Li, Shengnan Zhang, Ang Xiao, Jinyi Yu, Mingtian Yang, Fujin Lv, Mingyu Zhang, Gangping Hao, Guotong Chen, Liman Zhou, Fandong Kong, Guojun Pan

**Affiliations:** ^1^ State Key Laboratory of Crop Biology, College of Agronomy, Shandong Agriculture University, Tai’an, China; ^2^ College of Life Sciences, Shandong First Medical University, Shandong Academy of Medical Sciences, Tai’an, China; ^3^ Key Laboratory of Chemistry and Engineering of Forest Products, State Ethnic Affairs Commission, Guangxi Key Laboratory of Chemistry and Engineering of Forest Products, Guangxi Collaborative Innovation Center for Chemistry and Engineering of Forest Products, School of Chemistry and Chemical Engineering, Guangxi University for Nationalities, Nanning, China

**Keywords:** fungus, *Penicillium* sp. HFF16, indole-terpenoids, anti- diabetic activity, *Cynanchum bungei* Decne

## Abstract

Finding novel anti-diabetic compounds with effective suppression activities against hepatic glucagon response is urgently required for the development of new drugs against diabetes. Fungi are well known for their ability to produce new bioactive secondary metabolites. As part of our ongoing research, five new indole-terpenoids (**1**–**5**), named encindolenes D-H, were isolated from the fungus *Penicillium* sp. HFF16 from the rhizosphere soil of *Cynanchum bungei* Decne. The structures of the compounds were elucidated by spectroscopic data and ECD analysis. In the anti-diabetic activity assay, compounds **1–5** could inhibit the hepatic glucose production with EC_50_ values of 17.6, 30.1, 21.3, 9.6, and 9.9 *μ*M, respectively, and decrease the cAMP contents in glucagon-induced HepG2 cells.

## Introduction

**FIGURE 1:**
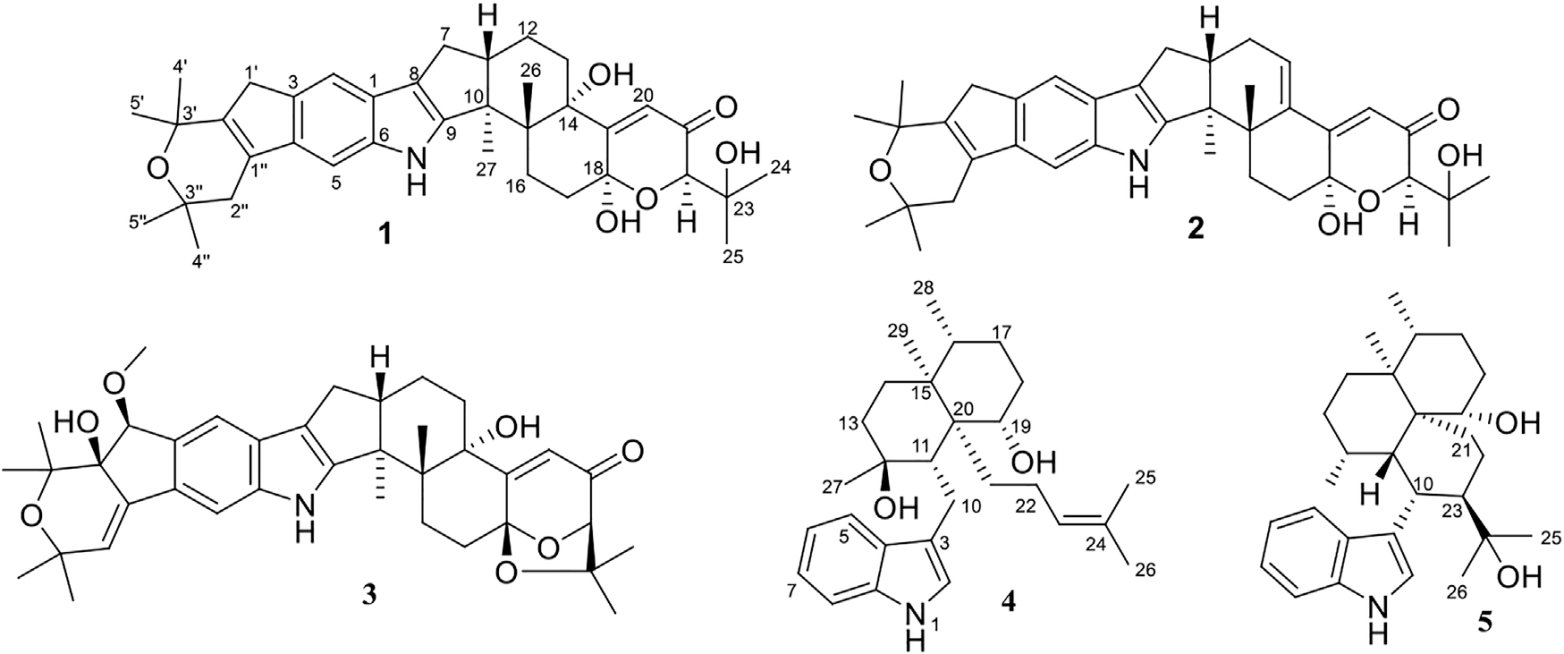
The chemical structures of compounds **1**–**5**.

Microorganism have been proven to be an important source of structurally novel and biologically active natural compounds, many of which have potential for drug development. In recent years, more and more attention has been paid to the study of active metabolites from fungi, of which paxilline-type indole-diterpenoids is well known for their diverse structures and bioactivities (Kong et al., 2019). Structurally and biosynthetically, paxilline-type indolediterpenes bear a common core structure derived from indole and geranylgeranyl diphosphate (GGPP), and further modifications, such as hydroxylation and prenylation, afforded other members of this family. The gene cluster *pax* was identified as the first biosynthetic gene cluster of this family (Tagami et al., 2013). Diabetes is a group of metabolic diseases characterized by hyperglycemia, which is caused by impaired peripheral glucose uptake and elevated hepatic glucose production (Unger and Cherrington, 2012; Jiang et al., 2021). Enhanced glucagon response is proposed to be responsible for increased hepatic glucose production; it is proposed that suppression of hepatic glucagon response may provide therapeutic advantages in diabetes management (Ozcan et al., 2012; Xiao et al., 2017). Therefore, finding novel and effective suppression of hepatic glucagon response anti-diabetic compounds is urgently required. The paxilline-type indole-terpenoids are one of the largest classes of fungal indole-terpenoids (Kong et al., 2019), many of which have significant bioactivities. In our ongoing search for bioactive metabolites from fungi (Pan GJ et al., 2021; Pan G et al., 2021), the secondary metabolites produced by *Penicillium* sp. HFF16 isolated from the rhizosphere soil of *Cynanchum bungei* Decne. from Mount Tai, China, were investigated, which resulted in the isolation and identification of five new indole-terpenoids with weak anti-inflammatory activities (Pan GJ et al., 2021). Subsequent chemical investigation on the same extract from *Penicillium* sp. HFF16 led to the identification of another five new indole-terpenoids (**1**–**5**) (Figure 1). All of the compounds exhibited moderate anti-diabetic effects on glucagon-stimulated cAMP accumulation and hepatic glucose production in HepG2 cells. Herein, the isolation, structural elucidation, and bioactivities of these compounds were described.

## Materials and Methods

### General Experimental Procedures

Optical rotations were measured on a JASCO P-1020 digital polarimeter, and UV spectra were measured on a Beckman DU 640 spectrophotometer. ECD data were collected using a JASCO J-715 spectropolarimeter. NMR spectra were recorded on a Bruckmercury Plus-400 or a JNM-ECZR-500 spectrometers with TMS as an internal standard. HRESIMS spectra were recorded with a Micromass Autospec -Uitima- TOF. Semi-preparative HPLC was carried out using an ODS column (YMC-pack ODS-A, 10 × 250 mm, 5 μm, 4 ml/min). Thin layer chromatography (TLC) and column chromatography (CC) were performed on plates precoated with silica gel GF_254_ (10–40 μm, Yantai Jiangyou Silicone Development Co., Ltd.).

### Fungal Material and Fermentation

The fungus *Penicillium* sp. HFF16 was isolated from the rhizosphere soil of *Cynanchum bungei* Decne., in Mount Tai, China in May 2020. After grinding, the sample (1.0 g) was diluted to 10^−2^ g/ml with sterile H_2_O, 100 μl of which was deposited on Bengal red medium (maltose 20 g, monosodium glutamate 10 g, glucose 10 g, yeast extract 3 g, corn pulp 1 g, mannitol 20 g, sodium chloride 0.3 g, potassium dihydrogen phosphate 0.5 g, agar 20 g per liter of tap water) plate containing chloramphenicol (200 μg/ml) as a bacterial inhibitor. A single colony was transferred onto another PDA plate and was identified according to its morphological characteristics and ITS gene sequences (Pan GJ et al., 2021). A reference culture of *Penicillium* sp. HFF16 maintained at −80°C is deposited in our laboratory. The isolate was cultured on plates of PDA medium at 28°C for 4 days. Plugs of agar supporting mycelium growth were cut and transferred aseptically to 7 × 250 ml Erlenmeyer flasks each containing 100 ml of liquid medium (potato 200 g, glucose 20 g per liter of tap water) and cultured at 28°C at 150 RPM for 3 days. The seed liquid was inoculated aseptically into 140 × 1,000 ml Erlenmeyer flasks each containing rice medium (80 g rice, 100 ml tap water) at 0.5% inoculation amount and incubated at room temperature under static conditions for 35 days.

## Extraction and Isolation

The cultures (11.2 kg) were then extracted into 40 L of EtOAc (ethyl acetate) by soaking overnight. The extraction was repeated for three times. The combined EtOAc extracts were dried under vacuum to produce 38.2 g of extract. The EtOAc extract was subjected to a silica gel VLC (vacuum column chromatography) column, eluting with a stepwise gradient of 0, 9, 11, 15, 20, 30, 50, and 100% EtOAc in petroleum ether (v/v), to give 7 fractions (Fr. 1−7). Fraction 3 (10.2 g) was applied to ODS silica gel with gradient elution of MeOH (CH_3_OH)-H_2_O (1:5, 2:3, 3:2, 4:1, 1:0) to yield five subfractions (Fr. 3-1–Fr. 3-5). Fr. 3-2 (1.03 g) was applied to ODS silica gel with gradient elution of MeCN-H_2_O (1:4, 2:3, 3:2, 4:1) to yield five tertiary fractions (Fr. 3-2-1–Fr. 3-2-5). Fr. 3-2-4 (92 mg) was purified using semi-prep HPLC (isocratic system 80% MeOH/H_2_O, v/v) to give compounds **4** (*t*_R_ 7.9 min; 4 mg) and **5** (*t*_R_ 11.8 min; 5.8 mg). Fraction 2 (2.3 g) was applied to ODS silica gel with gradient elution of MeOH-H_2_O (1:5, 2:3, 3:2, 4:1, 1:0) to yield five subfractions (Fr. 2-1–Fr. 2-5). Fr. 2-4 (66 mg) was purified using semi-prep HPLC (isocratic system 75% MeCN/H_2_O, v/v) to give compound **2** (*t*_R_ 22.36 min; 7.8 mg). Fr. 2-5 (124 mg) was purified using semi-prep HPLC (isocratic system 75% MeCN/H_2_O, v/v) to give compound **1** (*t*_R_ 21.58 min; 6.9 mg). Fraction 3 (2.13 g) was applied to ODS silica gel with gradient elution of MeOH-H_2_O (1:5, 2:3, 3:2, 4:1, 1:0) to yield eight subfractions (Fr. 3-1–Fr. 3-8). Fr. 3-6 (56 mg) was further purified using semi-prep HPLC (isocratic system 85% MeCN/H_2_O, v/v) to give compound **3** (*t*_R_ 7.6 min; 5.2 mg).

*Encindolene D* (**1)**: white powder; [*α*]25 D -6 (*c* 0.1, MeOH); UV (MeOH) *λ*_max_ (log *ε*): 307 (3.18), 239 (3.36) nm; ECD (MeOH) *λ*_max_ 218 (-11.34), 249 (+3.74), 269 (-2.26), 355 (-0.55) nm. ^1^H and ^13^C NMR data, Table 1; HRESIMS *m/z* 602.3464 [M + H]^+^ (calcd for C_37_H_48_NO_6_, 602.3476).

**TABLE 1:** The ^1^H (400 MHz) and ^13^C NMR (100 MHz) data of compounds **1–3** in CD_3_OD.

Position	1		2		3	
	*δ*_C_	*δ*_H_ (*J* in Hz)	*δ*_C_	*δ*_H_ (*J* in Hz)	*δ*_C_	*δ*_H_ (*J* in Hz)
1	125.1, C	7.39, s	124.8, C	7.37, s	127.8, C	7.43, s
2	114.8, CH		114.9, CH		116.1, CH	
3	136.0, C	7.11, s	136.2, C	7.14, s	135.8, C	
4	141.6, C		142.5, C		132.6, C	
5	102.9, CH		103.1, CH		105.0, CH	7.47, s
6	140.5, C	2.31, dd (11.6, 11.6)	140.9, C		143.2, C	
7	28.8, CH_2_		28.8, CH_2_	2.79, overlap	28.3, CH_2_	2.37, dd (11.4, 11.5)
		2.68, overlap		2.83, overlap		2.69, overlap
8	117.7, C		118.8, C		117.5, C	
9	153.5, C		149.6, C		156.0, C	
10	52.6, C		50.2, C		53.1, C	
11	51.3, CH	2.42, overlap	47.3, CH	2.86, m	50.1, CH	3.33, overlap
12	22.8, CH_2_	1.46, m	29.7, CH_2_	2.35, m	29.4, CH_2_	1.81, overlap
		1.87, m		2.26, m		2.02, overlap
13	34.3, CH_2_		134.1, CH	6.10, br s	33.9, CH_2_	1.91, overlap
						2.04, overlap
14	78.7, C		143.5, C		77.8, C	
15	44.9, C		44.7, C		40.9, C	
16	27.7, CH_2_	1.69, m	30.7, CH_2_	1.97, m	27.7, CH_2_	1.90, overlap
		2.53, m		2.40, m		2.62, m
17	37.4, CH_2_	1.71, m	35.4, CH_2_	2.14, m	22.3, CH_2_	1.72, m
		1.55, m		2.21, m		2.06, overlap
18	96.3, C		96.1, C		106.1, C	
19	163.4, C		164.5, C		172.2, C	
20	122.3, CH	5.67, s	122.9, CH	5.93, s	122.2, CH	6.06, s
21	200.9, C		201.8, C		199.4, C	
22	79.4, CH	4.27, s	80.0, CH	4.26, s	89.2, CH	4.30, s
23	73.8, C		74.4, C		79.4, C	
24	26.0, CH_3_	1.28, s	26.1, CH_3_	1.29, s	23.4, CH_3_	1.15, s
25	27.1, CH_3_	1.28, s	27.4, CH_3_	1.28, s	29.2, CH_3_	1.39, s
26	20.7, CH_3_	0.68	23.9, CH_3_	1.14, s	24.8, CH_3_	1.42, s
27	17.0, CH_3_	1.24, s	16.0, CH_3_	0.99, s	16.6, CH_3_	1.40, s
1′	37.6, CH_2_	3.33, s	37.7, CH_2_	3.31, overlap	91.5, CH	4.62, s
2′	143.4, C		143.7, C		81.6, C	
3′	76.0, C		76.1, C		79.5, C	
4′	31.5, CH_3_	1.43, s	31.5, CH_3_	1.42, s	23.9, CH_3_	1.41, s
5′	31.4, CH_3_	1.43, s	31.5, CH_3_	1.42, s	29.7, CH_3_	1.42, s
1″	134.0, C		133.3, C		132.6, C	
2″	35.9, CH_2_	2.43, s	36.1, CH_2_	2.44, s	122.2, CH	6.06, s
3″	73.8, C		73.8, C		74.3, C	
4″	30.5, C	1.33, s	30.7, C	1.32, s 1.32, s	32.8, CH_3_	1.42, overlap
5″	30.6, C	1.34, s	30.7, C		27.5, CH_3_	1.43, overlap
1′-OCH_3_					57.6, CH_3_	3.48, s

Encindolene E (2): white powder [α]25 D -205 (c 0.1, MeOH); UV (MeOH) λmax (log ε): 303 (3.33), 243 (3.27) nm; ECD (MeOH) λmax 221 (-4.62), 245 (+1.34), 282 (-15.45), 361 (-3.10) nm. 1H and 13C NMR data, Table 1; HRESIMS m/z 606.3172 [M + H]+ (calcd for C37H45NO5Na, 606.3190).

Encindolene F (3): white powder; [α]25 D +9 (c 0.1, MeOH); UV (MeOH) λmax (log ε): 334 (2.74), 268 (3.05) nm. 1H and 13C NMR data, Table 1; HRESIMS m/z 628.3274 [M - H]- (calcd for C38H46NO7, 628.3280).

Encindolene, Encindolene G (4): white powder; [α]25 D +11 (c 0.1, MeOH); UV (MeOH) λmax (log ε): 284 (2.28), 227 (2.96) nm. 1H and 13C NMR data, Table 2; HRESIMS m/z 422.3078 [M - H]- (calcd for C28H40NO2, 422.3065).

**TABLE 2:** The ^1^H (400 MHz) and ^13^C NMR (100 MHz) data of compounds **4** and **5** in CD_3_OD.

Position	4		5	
	*δ*_C_	*δ*_H_ (*J* in Hz)	*δ*_C_	*δ*_H_ (*J* in Hz)
2	123.7, CH	7.18, s	124.6, CH	7.25, s
3	119.6, C		117.9, C	
4	128.9, C		128.3, C	
5	119.4 CH	7.64, d (8.1)	118.6, CH	7.48, d (8.0)
6	119.2 CH	6.97, t (8.1)	119.9, CH	7.04, t (8.0)
7	121.9, CH	7.04, (8.1)	122.6, CH	7.11, t (8.0)
8	112.0, C	7.29, d (8.1)	112.5, CH	7.37, d (8.0)
9	137.7, C		137.7, C	
10	22.8, CH_2_	3.15, dd (15.0, 6.1)	34.2, CH	3.78, dd (12.7, 5.4)
		2.96, br d (15.0)		
11	52.0, CH	2.77, m	42.2, CH	2.16, dd (5.4, 5.4)
12	75.6, C			
13	38.1, CH_2_	1.86, m	25.0, CH_2_	2.05, m
		1.53, m		1.83, m
14	31.7, CH_2_	1.59, m	29.3, CH_2_	0.94, m
		1.38, m		1.60, m
15	41.2, C		40.3, C	
16	32.1, CH	2.35, m	32.3, CH	2.09, m
17	26.5, CH_2_	1.02, m	26.7, CH_2_	1.78, m
		1.57, overlap		1.27, m
18	30.5, CH_2_	0.96, m	30.7, CH_2_	1.82, m
		1.36, overlap		2.08, m
19	70.0, CH	4.25, dd (2.7, 2.7)	69.3, CH	4.76, br s
20	49.0, C		44.9, C	
21	32.0, CH_2_	2.14, m	29.2, CH_2_	1.17, m
		1.60, m		1.60, m
22	26.9, CH_2_	2.42, m	23.9, CH_2_	1.70, m
				1.88, m
23	127.8, CH	5.18, m	46.1, CH	2.57, ddd (12.7, 12.0, 6.7)
24	131.3, C		76.6, C	
25	26.0, CH_3_	1.74, s	26.8, CH_3_	1.06, s
26	18.1, CH_3_	1.73, s	29.0, CH_3_	1.06, s
27	24.6, CH_3_	1.44, s	22.2, CH_3_	1.38, d (7.4)
28	16.7, CH_3_	0.77, d (6.6)	16.3, CH_3_	0.75, d (6.9)
29	18.7, CH_3_	0.93, s	18.7, CH_3_	0.97, s

*Encindolene H (* ***5)***: white powder; [*α*]25 D -28 (*c* 0.1, MeOH); UV (MeOH) *λ*_max_ (log *ε*): 285 (2.68), 226 (3.15) nm. ^1^H and ^13^C NMR data, Table 2; HRESIMS *m/z* 422.3059 [M - H]^-^ (calcd for C_28_H_40_NO_2_, 422.3065).

### Measurement of Cell Viability Assay

HepG2 cells (a cell line of human hepatoma, from the Type Culture Collection of the Chinese Academy of Sciences) were cultured in DMEM supplemented with 10% FBS, 100 μg/ml of streptomycin, and 100 U/ml of penicillin at 37°C in a 5% CO_2_ atmosphere. Cell viability was assessed by the MTT method (Pan GJ et al., 2021). HepG2 cells were seeded in a 96-well plate and treated with 100 nM glucagon (Novo Nordisk, Denmark) and various concentrations of test compounds (1–100 μM) for 24 h. After that, MTT solution (10 μl) was added and incubated at 37°C for 4 h. The purple crystals were dissolved with dimethylsulfoxide (150 μl) added, and the absorbance value was measured by a microplate reader at 570 nm.

## Glucose Output

HepG2 cells were maintained in DMEM medium with 10% FBS. After attachment, the media was replaced with Krebs-Ringer HEPES buffer to fast the cells for 2 h. Then, the cells were cultured with glucose out media supplemented with 10 mM pyruvate, 100 nM glucagon, or metformin and the indicated compounds (1, 5, 10, 50, and 100 μM). After 6 h, the cell supernatant was collected for glucose analysis.

### Measurement of cAMP Production

HepG2 cells were pretreated with the test compounds and stimulated with 100 nM glucagon for 4 h. The cAMP production in culture medium was calculated by commercial kit (Xiao et al., 2017). All data were expressed as the mean ± SD from at least three independent experiments.

## Results and Discussion

### Structure Elucidation of Compounds

**FIGURE 2:**
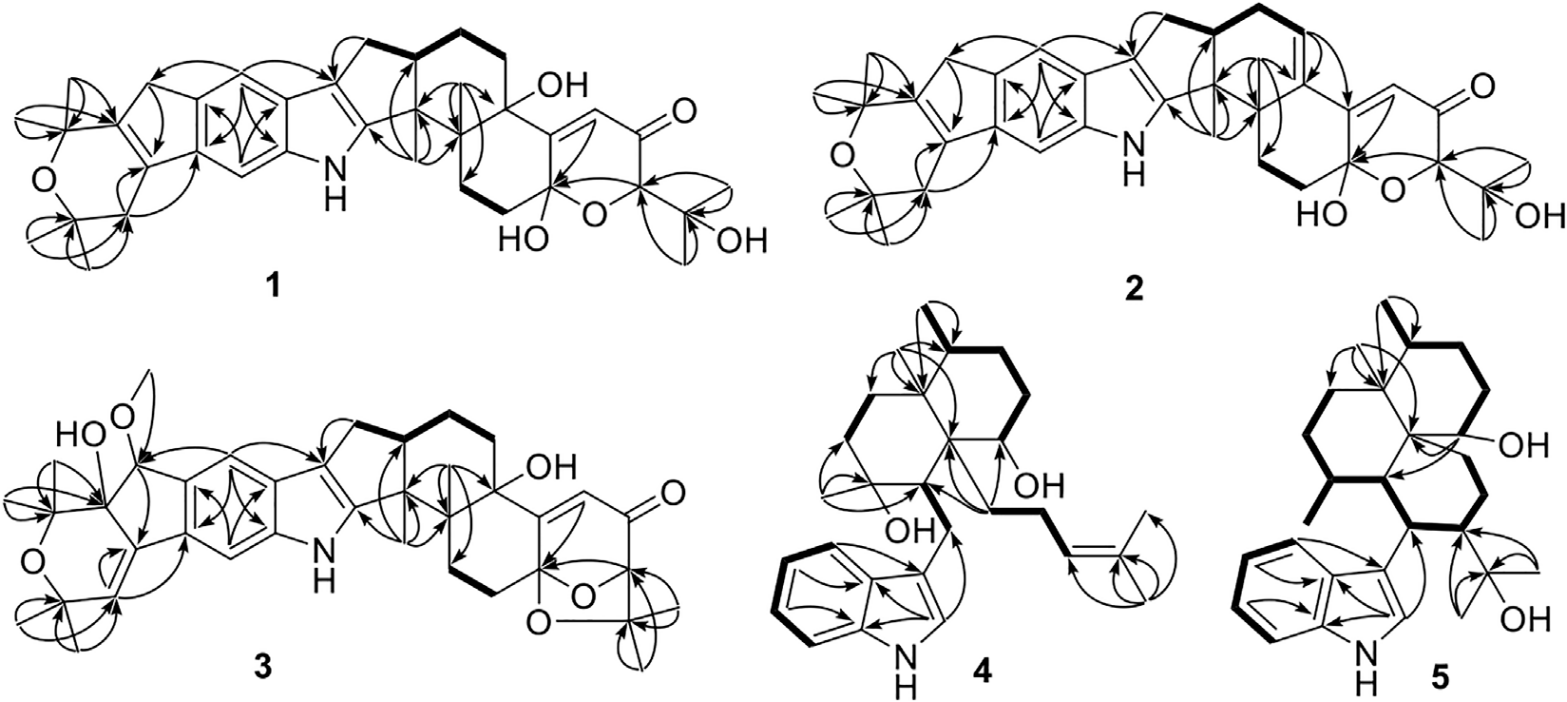
Selected HMBC and COSY correlations of **1**–**5**.

**FIGURE 3:**
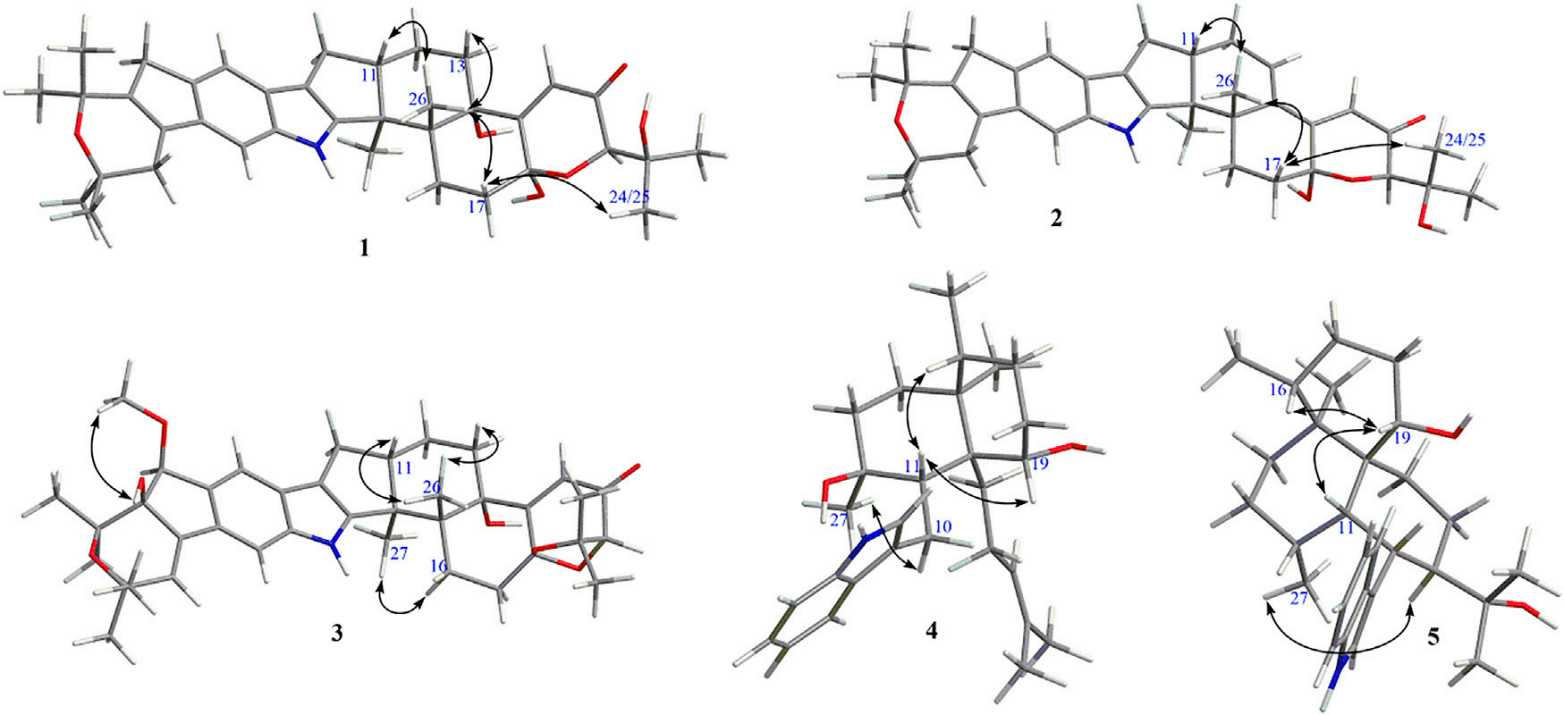
Selected ROESY correlations of **1**–**5**.

**FIGURE 4:**
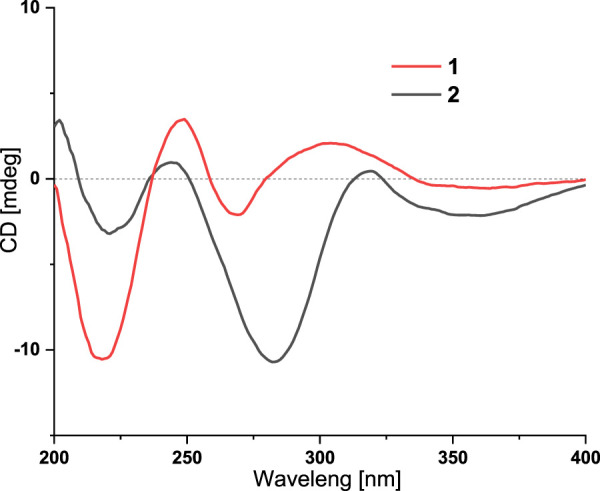
The experimental ECD spectra of **1** and **2**.

Compound **1** was assigned the molecular formula C_37_H_47_NO_6_ by HRESIMS. The double-bond equivalents of **1** were calculated to be 15. The ^13^C and HSQC NMR spectra (Table 1) of **1** revealed a total of 37 carbons including one ketone carbonyl, eight aromatic carbons (two protonated) attributed to one indole moiety, four olefinic carbons with one protonated, six oxygenated carbons with one protonated, seven sp^3^ methylenes, one sp^3^ non-oxygenated methine, two sp^3^ non-oxygenated quaternary carbons, and eight methyls. The above data were quite similar to those of pyrapaxilline (Matsui et al., 2014), a previous reported indole-diterpene with an additional dihydropyran ring. The main difference between them was the replacement of the oxygenated methine CH-18 in pyrapaxilline by a dioxygenated quaternary carbon at *δ*_C_ 96.3 in **1**, suggesting the presence of a hydroxyl at C-18. HMBC correlation from H-20 to C-18 further confirmed this deduction (Figure 2). The relative configuration of **1** was assigned by analysis of its ROESY spectrum (Figure 3), which showed correlations of H-11/H_3_-26/H-17/H-24 (25) and H_3_-26/H-13. The experimental ECD spectrum (Figure 4) of **1** showed negative Cotton effects (CEs) around 220, 260, and 350 nm, and positive ones around 250 and 300 nm, respectively (Figure 4), which was very similar to that for encindolene A (Pan GJ et al., 2021), an analogue isolated from the same fungus. This led to the assignment of the absolute configurations of **1** as shown in Figure 1.

Compound **2** was obtained as a white powder, and its molecular formula was determined as C_37_H_45_NO_5_ according to the HRESIMS data, with a molecule of H_2_O less than **1**. The NMR data of **2** were also quite similar to those of **1**. However, detailed comparison of the NMR data between them revealed that signals for the hydroxylated non-protonated carbon C-14 and the CH_2_-13 methylene in the NMR spectra of **1** were replaced by signals for a tri-substituted double bond in those of **2**. The location of this double bond at C-13/C-14 was revealed by COSY correlations (Figure 2) of H_2_-7/H-11/H_2_-12/H-13 and HMBC correlations (Figure 2) from H_3_-26 to C-14. Thus, **2** was determined to be a C-14/C-13 dehydrated derivative of **1**. The relative configuration of **2** was assigned to be the same as that of **1** based on analysis of the ROESY data (Figure 3). The absolute configurations of **2** were also assigned as shown in Figure 1 by a comparison of its ECD spectrum with that of **1** (Figure 4), which showed great similarity.

The molecular formula of compound **3** was established as C_38_H_47_NO_7_ by HRESIMS, with one more degree of unsaturation compared to **3**. The NMR spectra of **3** were closely related to those of **2**, indicating that **3** was also an indolediterpene bearing an additional substituted dihydropyran ring linked with the indole unit. The carbon skeleton of **3** was assigned the same as that of **2** by analysis of the 2D NMR data (Figure 2). However, in the HMBC spectrum of **3**, correlations from H_3_-4″ and H_3_-5″ to C-3″ and C-2″ at *δ*_C_ 122.2, as well as from H-2″ to C-1″ and C-4, suggested the presence of the C-1''/C-2″ double bond. HMBC correlations from H_3_-4′ and H_3_-5′ to C-2′ at *δ*_C_ 81.6 indicated the location of a hydroxyl group at C-3'. HMBC correlations from both H-2 and the methoxy protons at *δ*_H_ 3.48 to C-1′ at *δ*_C_ 91.5 suggested the presence of a methoxy at C-1'. The characteristic chemical shifts of the two oxygenated carbons C-18 and C-23 (*δ*_C_ 106.1 and 79.4), together with the molecular formula, suggesting the linkage of C-18/O/C-23. In the ROESY spectrum (Figure 3), correlation of H-11/H_3_-26 indicated the same orientation of these protons, while correlation of H-16/H_3_-27 suggested that they were on the face opposite to H_3_-26. In the ROSEY spectrum collected in DMSO-*d*_6_, correlation between the protons of OH-2′ and 1′-OCH_3_ indicated their same orientation.

The molecular formula of compound **4** was established as C_28_H_41_NO_2_ by HRESIMS. The double-bond equivalent of **4** was calculated to be nine. The HSQC spectrum displayed the typical pattern of a 3-substituted indole moiety, seven sp3 methylenes, five methyls, three sp^3^ methines with one oxygenated, three sp^3^ non-protonated carbons with one oxygenated, and one tri-substituted double bond. These data were nearly identical to those for penicilindole A (Zheng et al., 2018), and the main difference between them was the chemical shift for C-13, which was *δ*_C_ 47.7 for penicilindole A, while *δ*_C_ 52.0 for **4.** Detailed analysis of the HMBC and COSY data (Figure 2) for **4** revealed that it bears the same planar structure as that of penicilindole A. Similar to that of penicilindole A, correlations of H_3_-27/H-10/H-19 and H-11/H-16 were observed in the ROESY spectrum of **4**, leading to the assignment of the relative configurations for all of the chiral carbons except for C-27 (Figure 3). However, the absence of the correlation between H_3_-27 and H-11 in the ROESY data of **4** indicated their *trans* relationship, which was different from that of penicilindole A. Thus, compound **4** was determined to be 27-*epi*- penicilindole A.

Compound **5** was obtained as a white powder, and its molecular formula was determined as C_28_H_41_NO_2_ according to the HRESIMS data, with nine degrees of unsaturation. Analysis of the NMR spectra of **5** also revealed a 3-substituted indole moiety. Besides, five methyls, six sp^3^ methylenes, six sp^3^ methines with one oxygenated, and three sp^3^ non-protonated carbons with one oxygenated were also observed. The above data were comparable to those reported for 10,23-dihydro-24,25-dehydroaflavinine (Mark et al., 1989), with the main differences being the replacement of the signals for the methylene in 10,23-dihydro-24,25-dehydroaflavinine by one methyl and one oxygenated sp^3^ non-protonated carbon in **5**. In the HMBC spectrum of **5** (Figure 2), correlations from both H_3_-25 and H_3_-26 to C-24 at *δ* 76.6 and C-23 were observed. These data suggested that the C-24/C-25 double bond in 10,23-dihydro-24,25-dehydroaflavinine was hydrated in **5.** The remaining structure of **5** was determined to be the same as that of 10,23-dihydro-24,25-dehydroaflavinine by analysis of the 2D NMR data. The relative configuration of **5** was determined by ROSESY correlations (Figure 4) of H-11/H-19/H-16 and H_3_-27/H-23.

Until now, more than 100 paxilline-type indole-terpenoids have been reported. These compounds showed multiple activities, including anti-H1N1 (Fan et al., 2013), antibacterial (Xu et al., 2019), cytotoxic, ion channel antagonistic (Sheehan et al., 2009), and PTP1B inhibitory activities. However, the anti-diabetic activities of these compounds have not been reported.

### Anti-Diabetic Activity Assay

**FIGURE 5:**
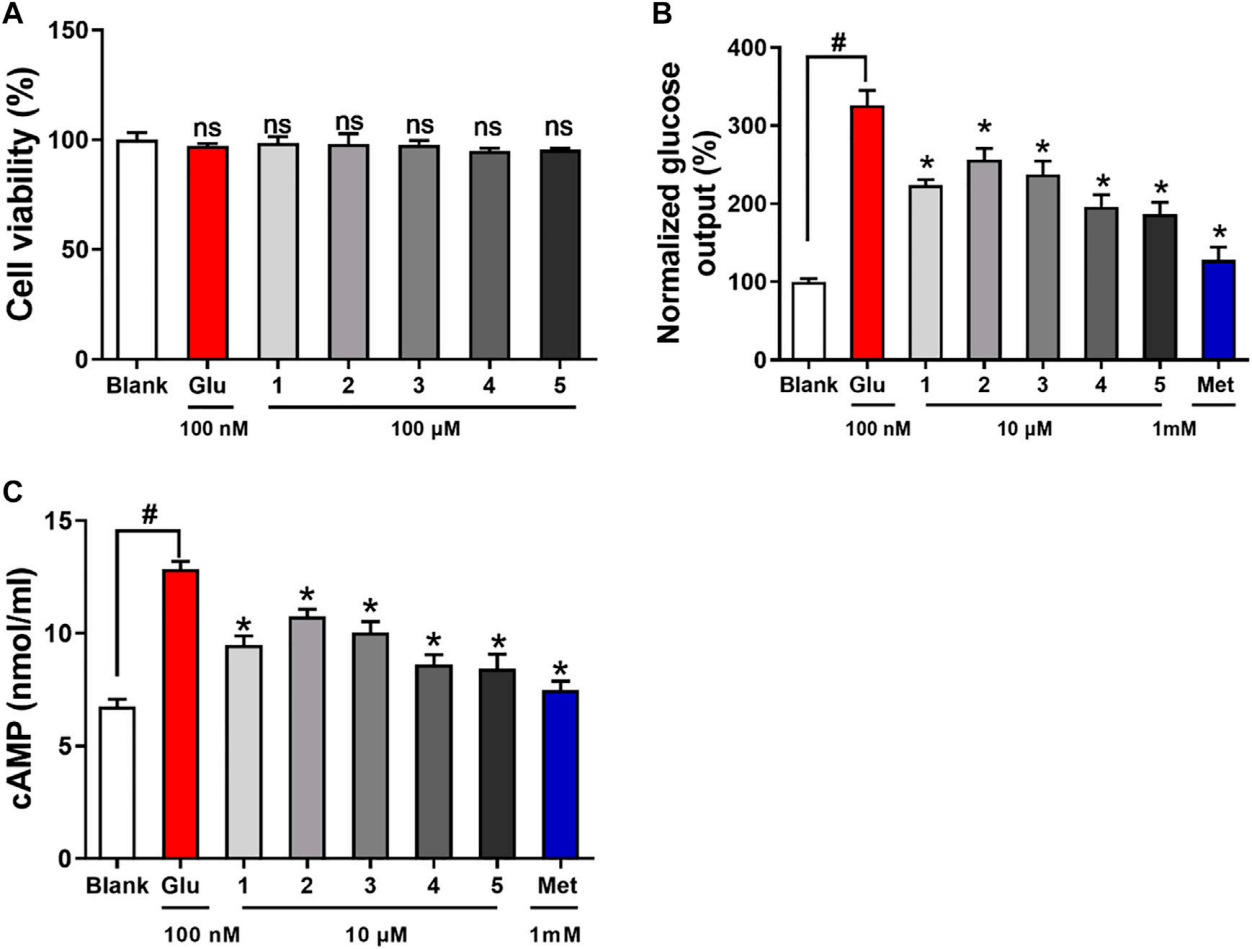
Viability and anti-diabetic effects of compounds **1–5** against HepG2 cells. **(A)**: cell viability **(B)**: hepatic glucose production level; **(C)**: cAMP contents in HepG2 cells treated with glucagon (Glu, 100 nM). ^ns^ *p*> 0.05 vs. Blank, *p < 0.05 vs. Glu, ^#^ *p* < 0.05 vs. Blank.

Compounds **1–5** were nontoxic against HepG2 cells by MTT assay at the concentration of 100 μM (Figure 5A). Excessive hepatic glucose production was considered to be a key for the onset of diabetes (Liao et al., 2021). The hepatic glucose production in response to all the compounds was evaluated, and EC50 values were used to assess their potencies. Glucagon challenge increased hepatic glucose production in HepG2 cells, whereas compounds **1–5** inhibited hepatic glucose production, with EC_50_ values of 17.6, 30.1, 21.3, 9.6, and 9.9 μM, respectively, while 1.9 μM for the positive control metformin. cAMP is a second messenger in response to glucagon and responsible for the initiation of cascade signaling of hepatic glucose production. Glucagon stimulation increased cAMP contents in cells (Figures 5B,C). Compound **1–5** treatment suppressed cAMP accumulation, with **4** showing the strongest effect. The results suggested that compounds **1–5** inhibited hepatic glucose production by suppressing hepatic glucagon response.

## Conclusion

In summary, from the fungus *Penicillium* sp. HFF16, five new indole-terpenoids were isolated and identified. These compounds could inhibit cAMP accumulation and hepatic glucose production, without affecting the cell viability in glucagon-stimulated HepG2 cells. Among them, compound **4** showed the strongest effect, which showed its potential in the development of new anti-diabetic drugs.

## Data Availability

The raw data supporting the conclusions of this article will be made available by the authors, without undue reservation.
