# Supplementary material for: Anti-Diabetic Indole-Terpenoids From Penicillium sp. HFF16 Isolated From the Rhizosphere Soil of Cynanchum bungei Decne
Source: Front Chem. 2022 Feb 8;9:792810. doi: 10.3389/fchem.2021.792810 (PMC8861352; doi:10.3389/fchem.2021.792810)

Supplementary Material

Anti-diabetic Indole-terpenoids from *Penicillium* sp. HFF16 isolated from the rhizosphere soil of *Cynanchum bungei* Decne.

Na Xiao,[1] Yiru Xu,[3] Xinru Zhang,[3] Haonan Li,[3] Shengnan Zhang,[3] Ang Xiao,[3] Jinyi Yu,[3] Mingtian Yang,[3] Fujin Lv,[3] Mingyu Zhang,[3] Gangping Hao,[3] Guotong Chen,[2] Liman Zhou,[2] Fandong Kong,[2*] Guojun Pan [3 *]

[1] State Key Laboratory of Crop Biology, college of Agronomy, Shandong Agriculture University, Tai'an, Shandong 271018, China.

[2] Key Laboratory of Chemistry and Engineering of Forest Products, State Ethnic Affairs Commission, Guangxi Key Laboratory of Chemistry and Engineering of Forest Products, Guangxi Collaborative Innovation Center for Chemistry and Engineering of Forest Products, School of Chemistry and Chemical Engineering, Guangxi University for Nationalities, Nanning 530006, China.

[3] College of Life Sciences, Shandong First Medical University ＆ Shandong Academy of Medical Sciences, Tai'an, Shandong 271000, China.

NMR and HRESIMS spectra of compound 1……………………………….2

NMR and HRESIMS spectra of compound 2……………………………….6

NMR and HRESIMS spectra of compound 3…………..…………………10

NMR and HRESIMS spectra of compound 4…………………...…………...15

NMR and HRESIMS spectra of compound 5…………………...…………...19

NMR and HRESIMS spectra of compound 1

^{1}H-NMR spectrum of 1 in CD_3OD

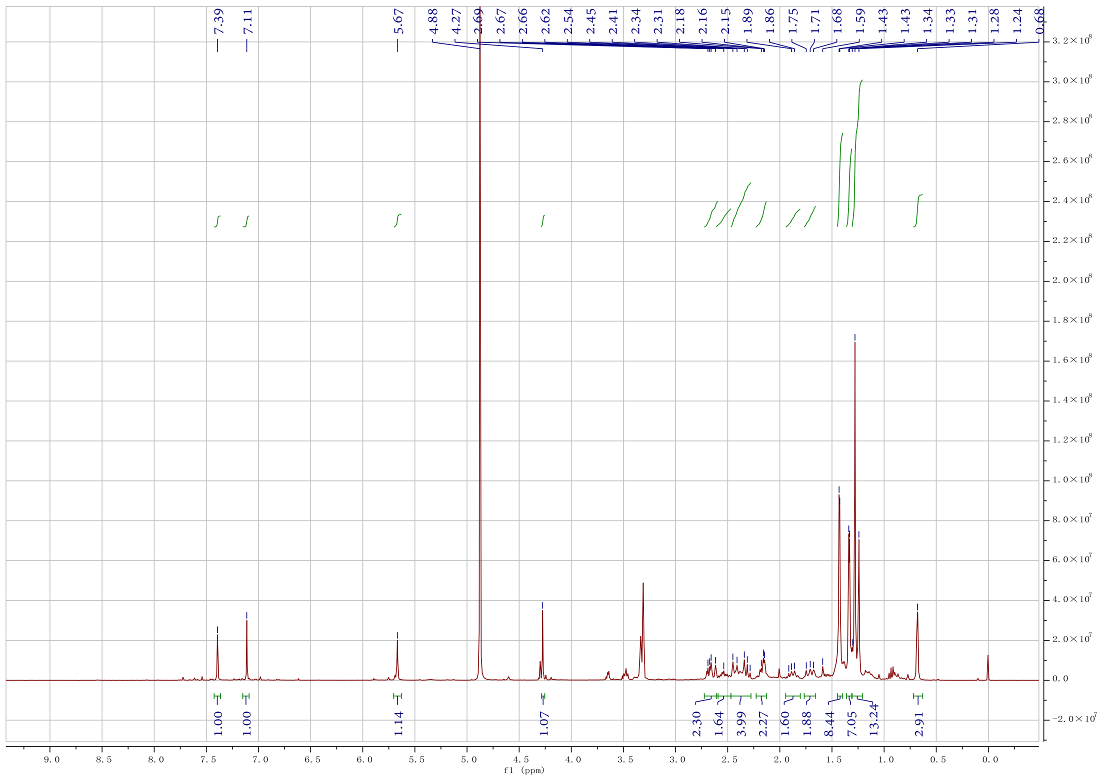

^{13}C-NMR spectrum of 1 in CD_3OD

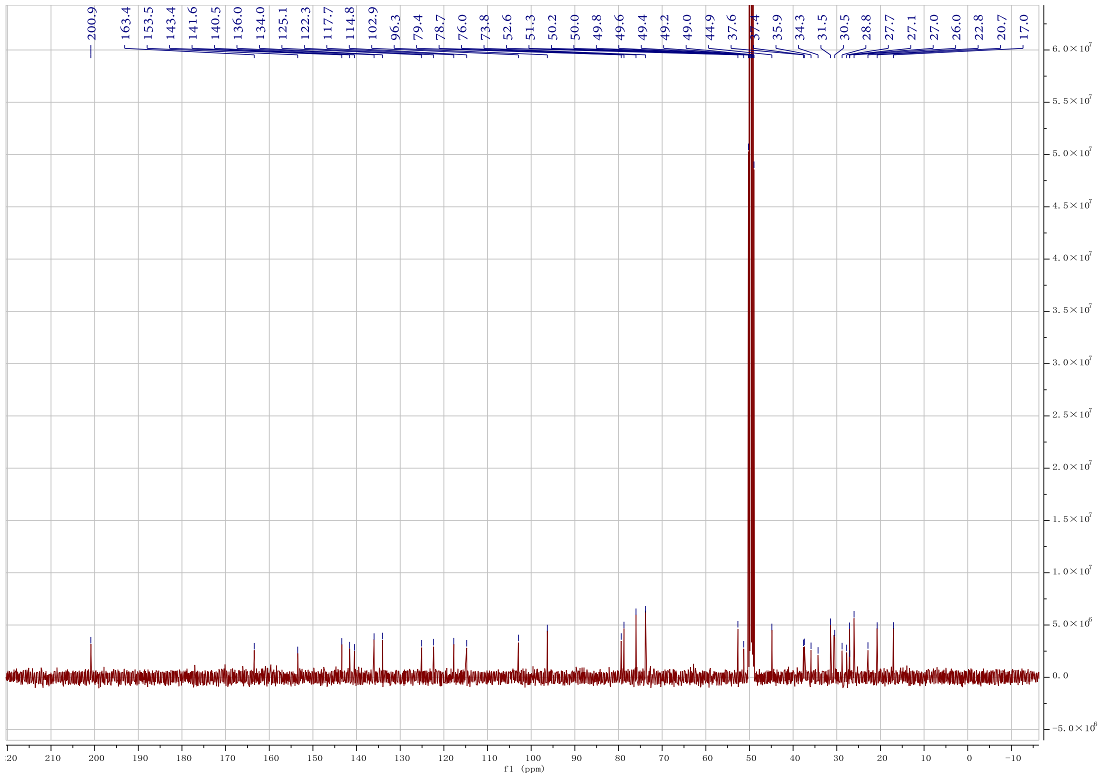

DEPT spectrum of 1 in CD_3OD

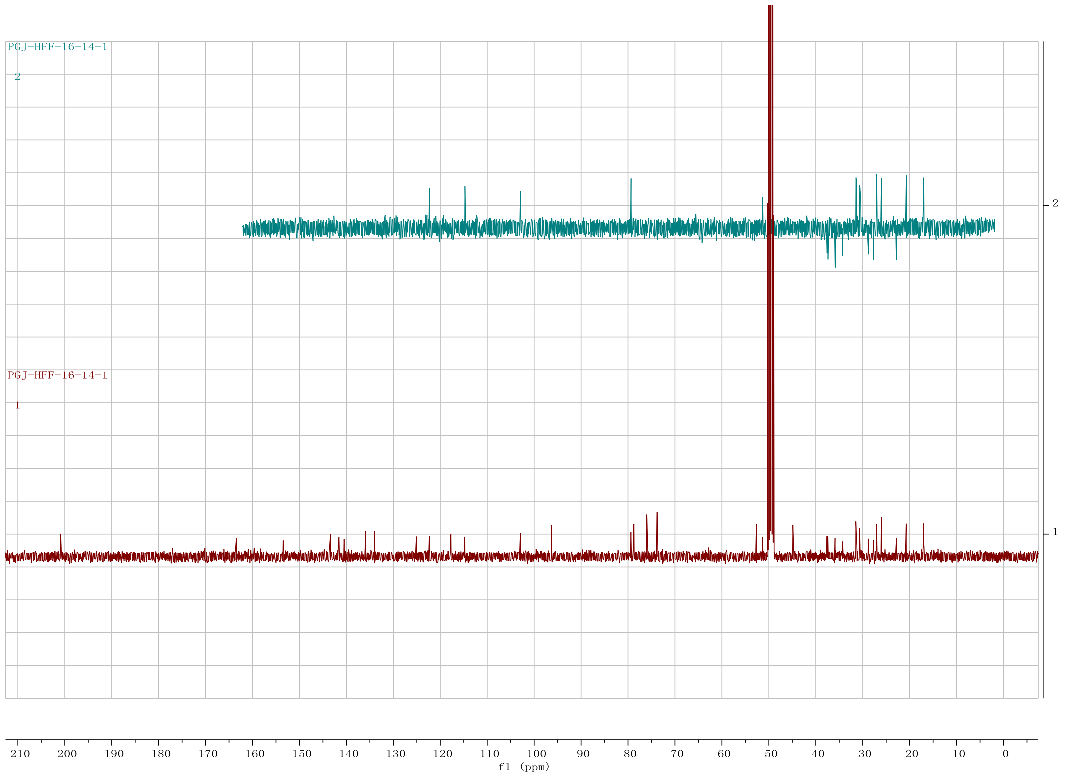

HSQC spectrum of 1 in CD_3OD

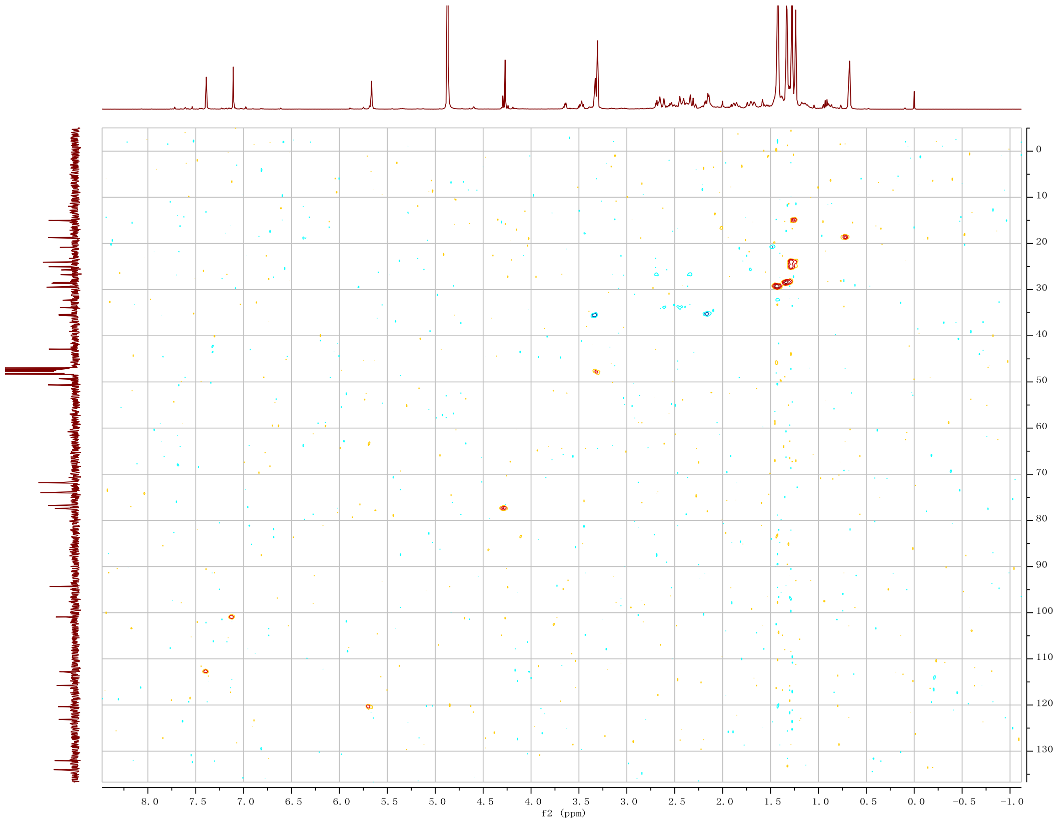

^{1}H-^{1}H COSY spectrum of 1 in CD_3OD

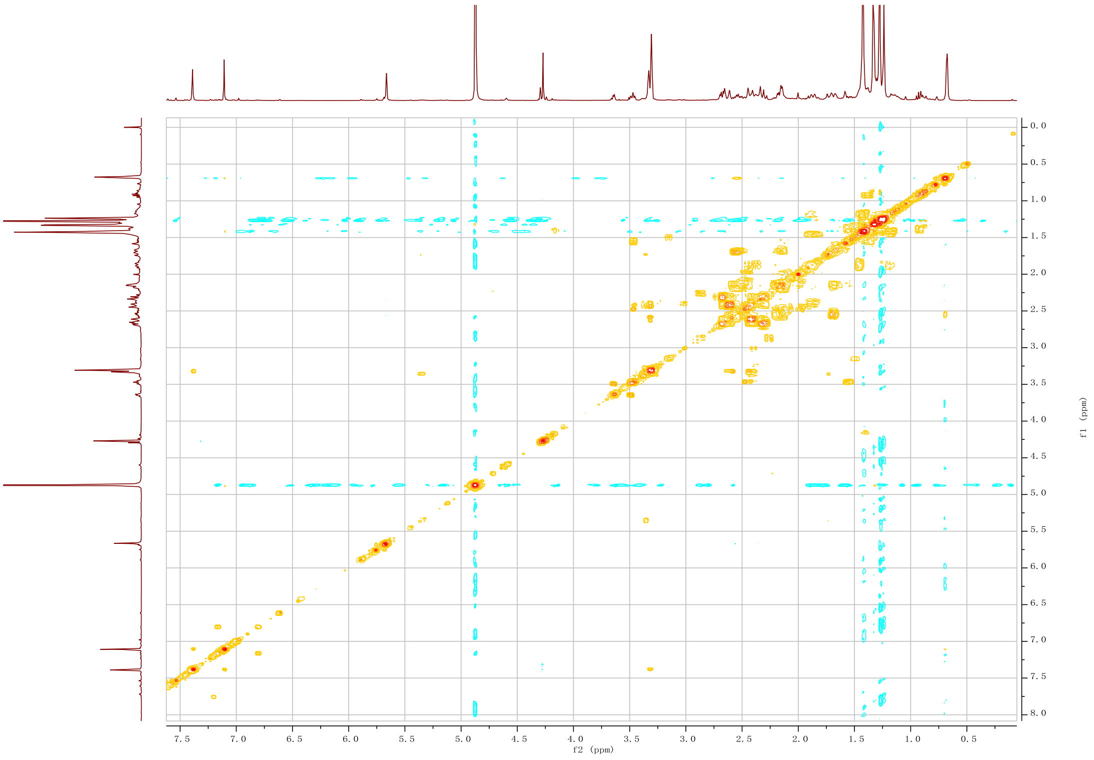

HMBC spectrum of 1 in CD_3OD

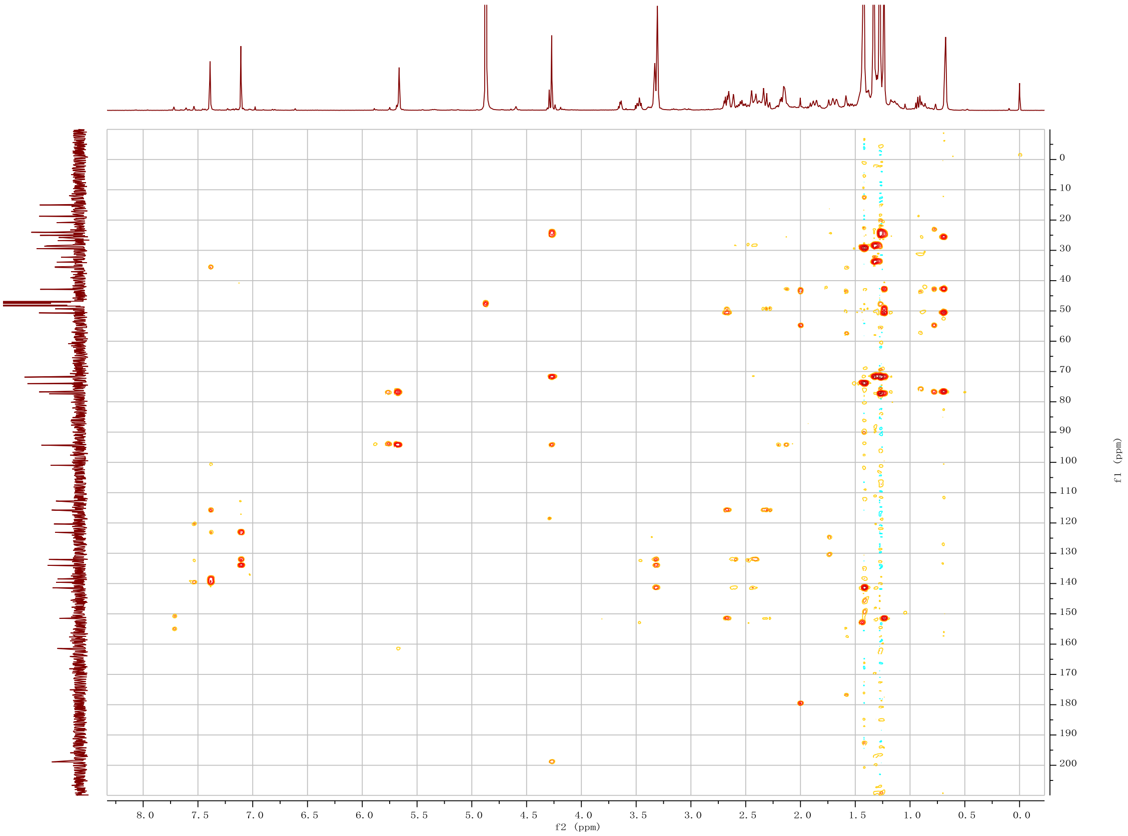

ROESY spectrum of 1 in CD_3OD

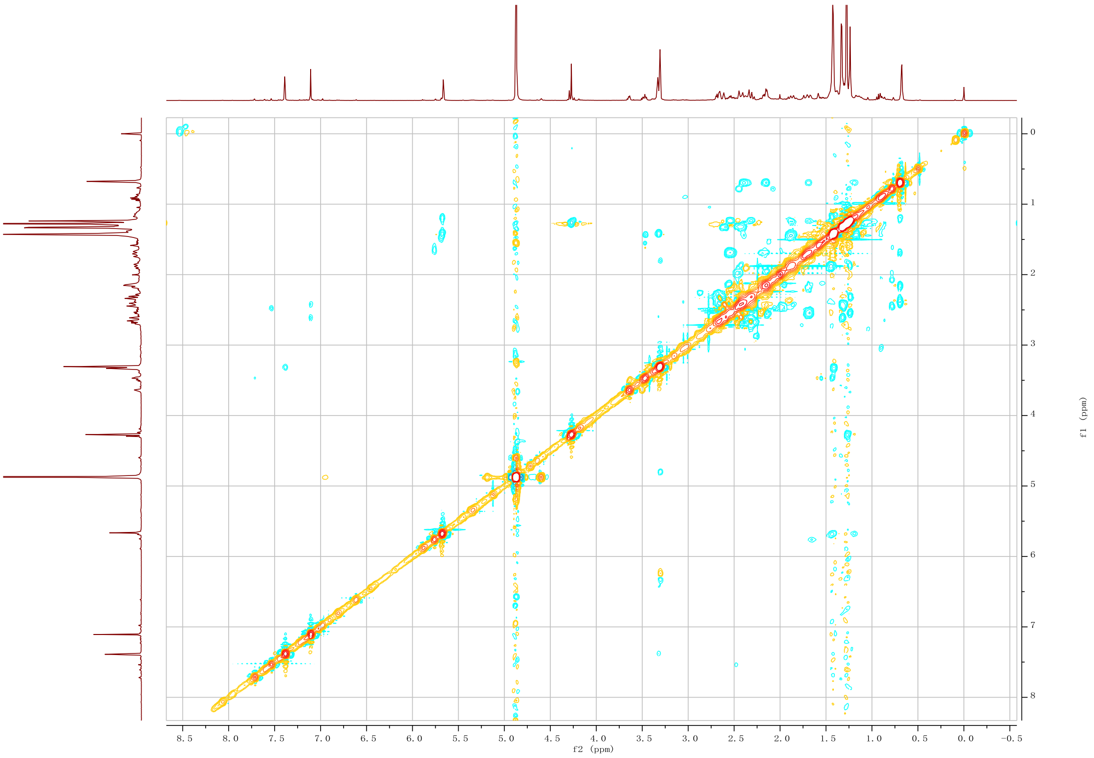

HRESIMS spectrum of 1

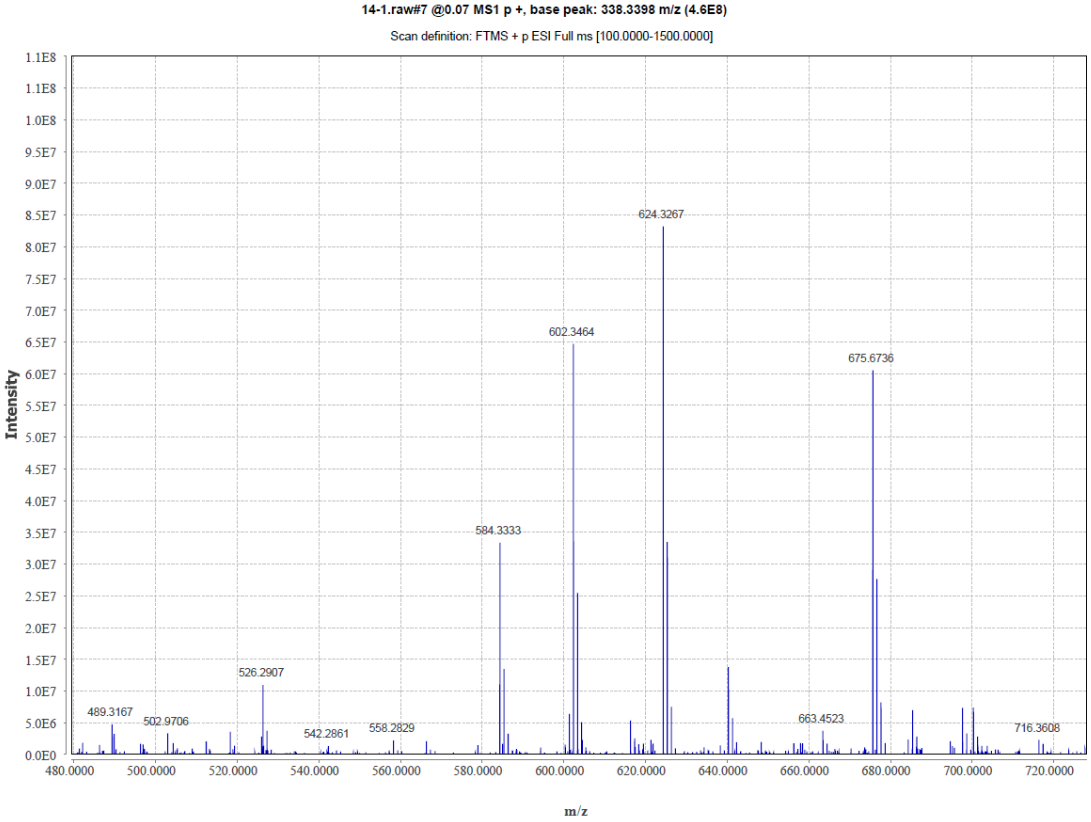

NMR and HRESIMS spectra of compound 2

^{1}H-NMR spectrum of 2 in CD_3OD

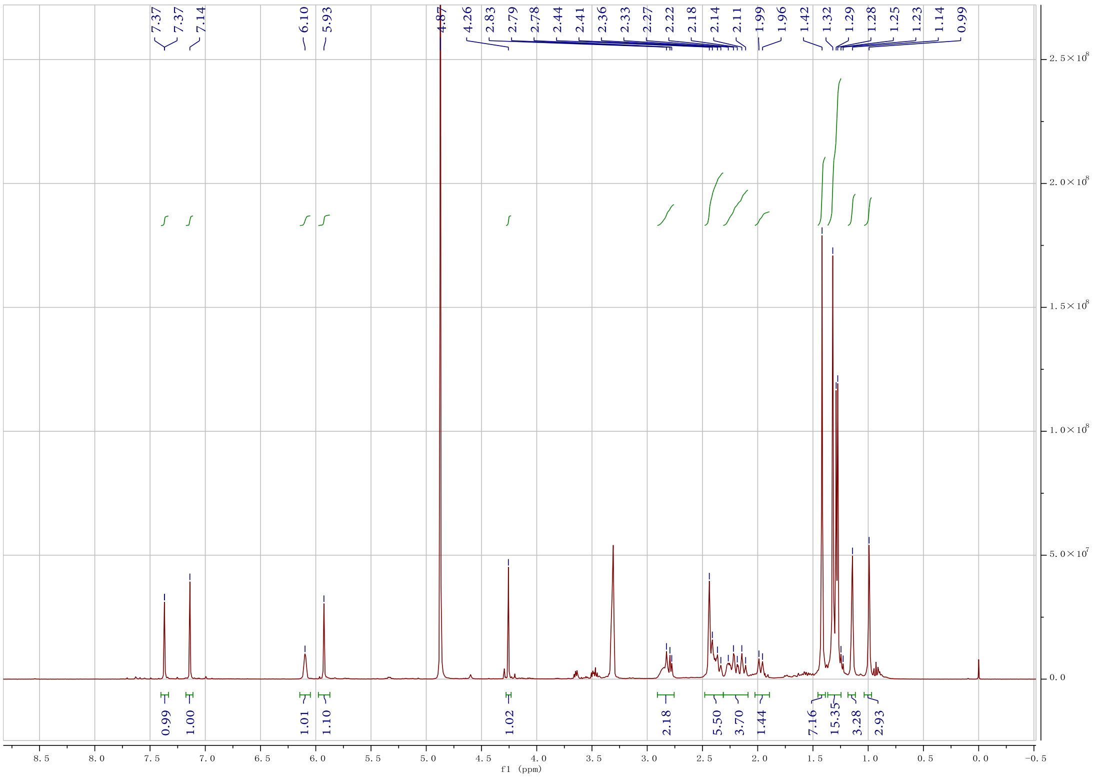

^{13}C-NMR spectrum of 2 in CD_3OD

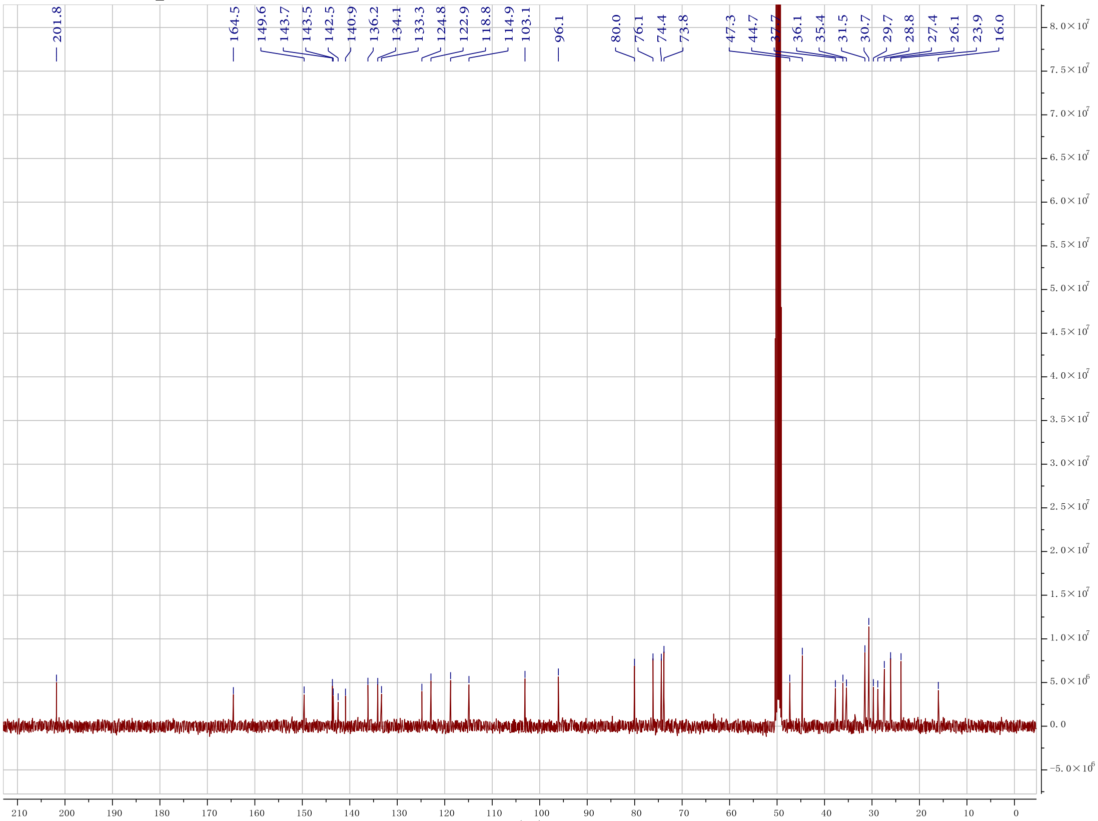

DEPT spectrum of 2 in CD_3OD

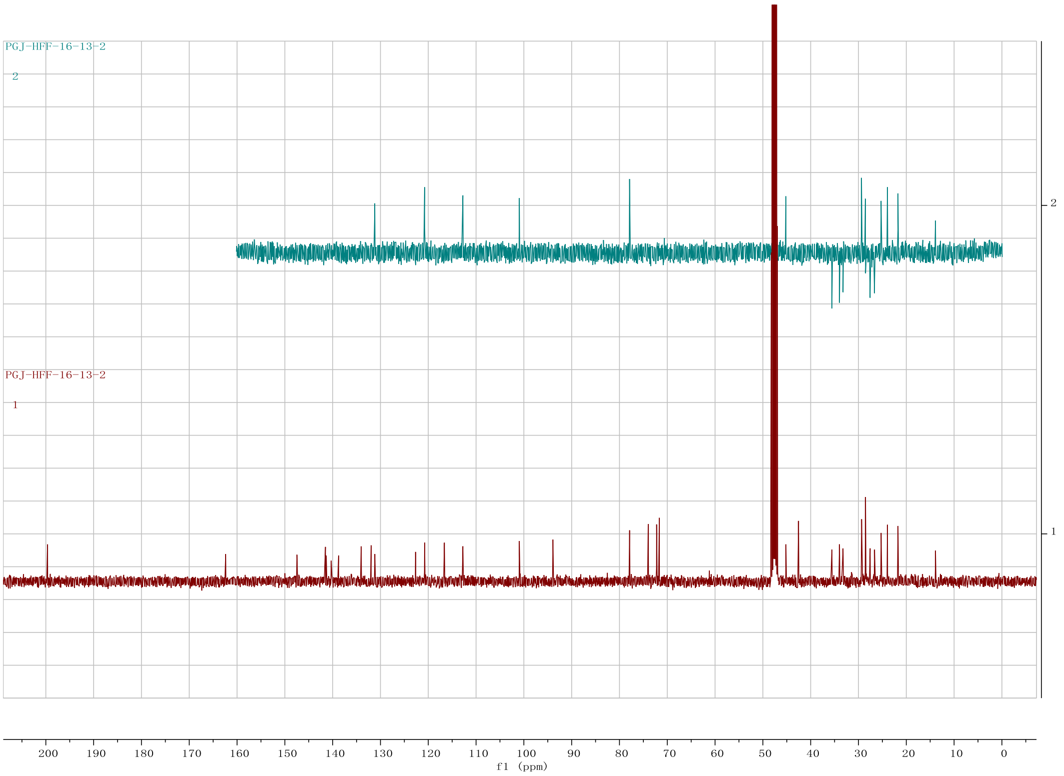

HSQC spectrum of 2 in CD_3OD

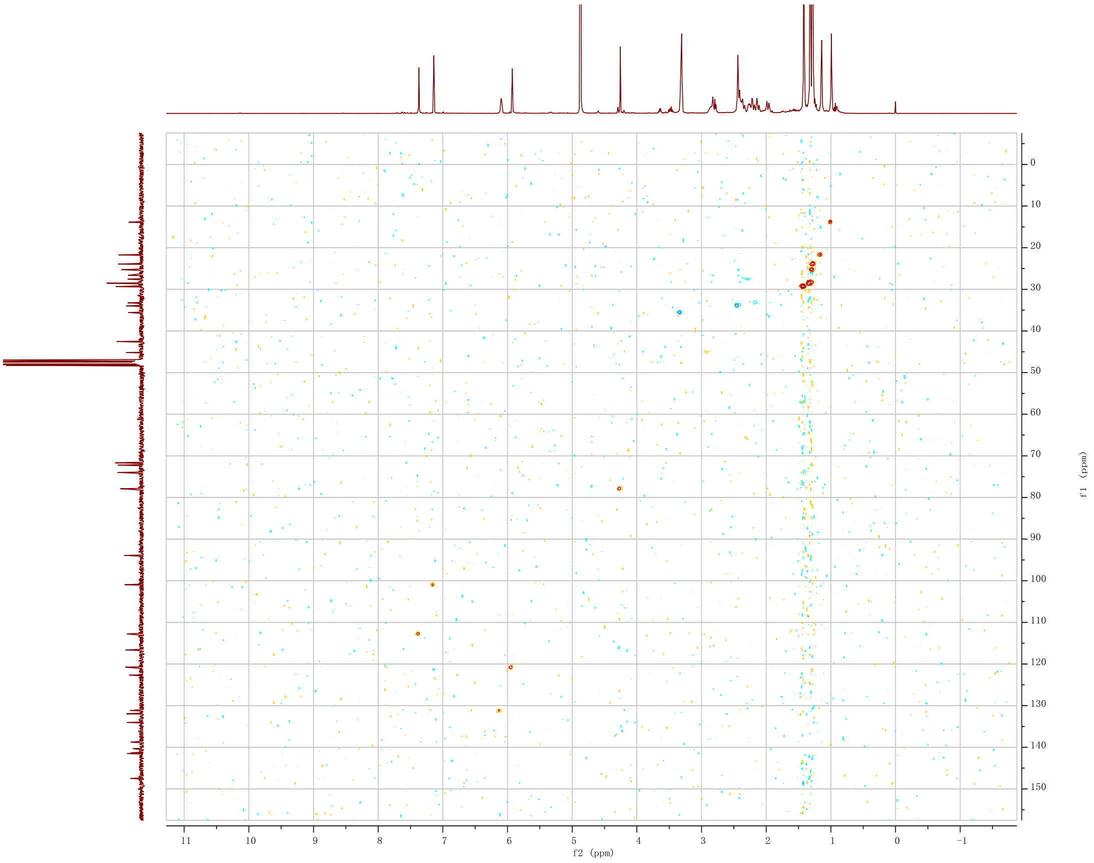

^{1}H-^{1}H COSY spectrum of 2 in CD_3OD

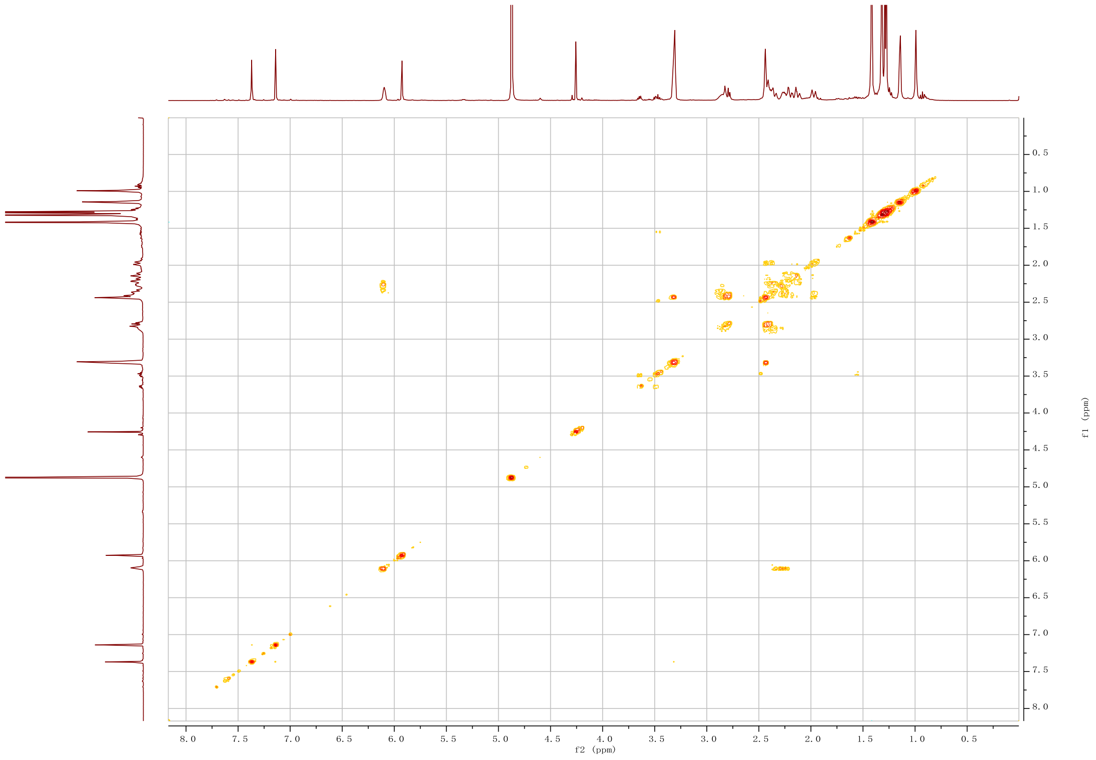

HMBC spectrum of 2 in CD_3OD

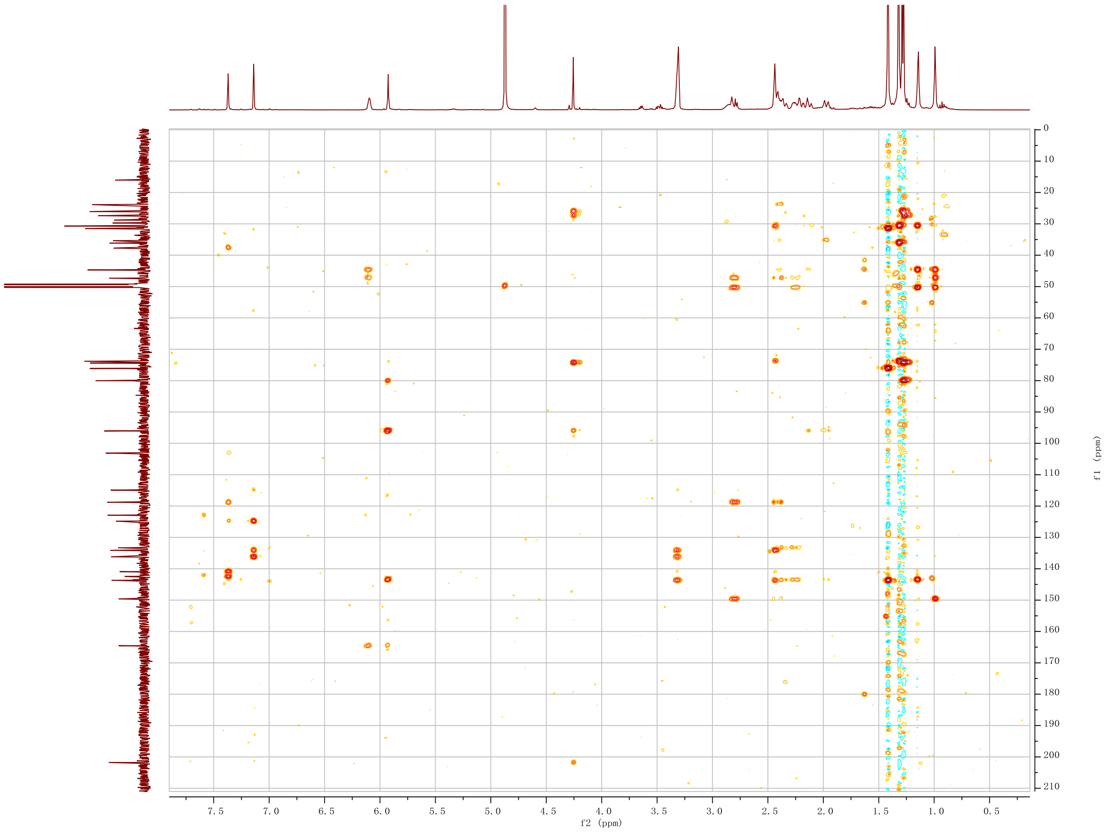

ROESY spectrum of 2 in CD_3OD

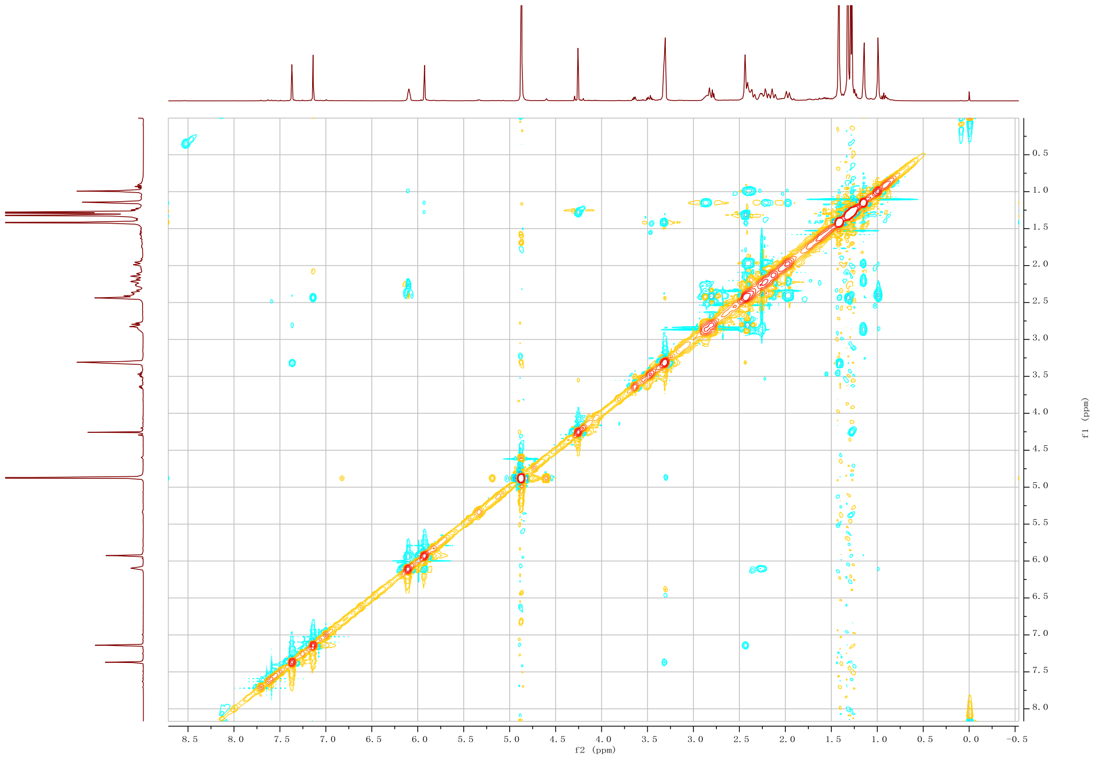

HRESIMS spectrum of 2

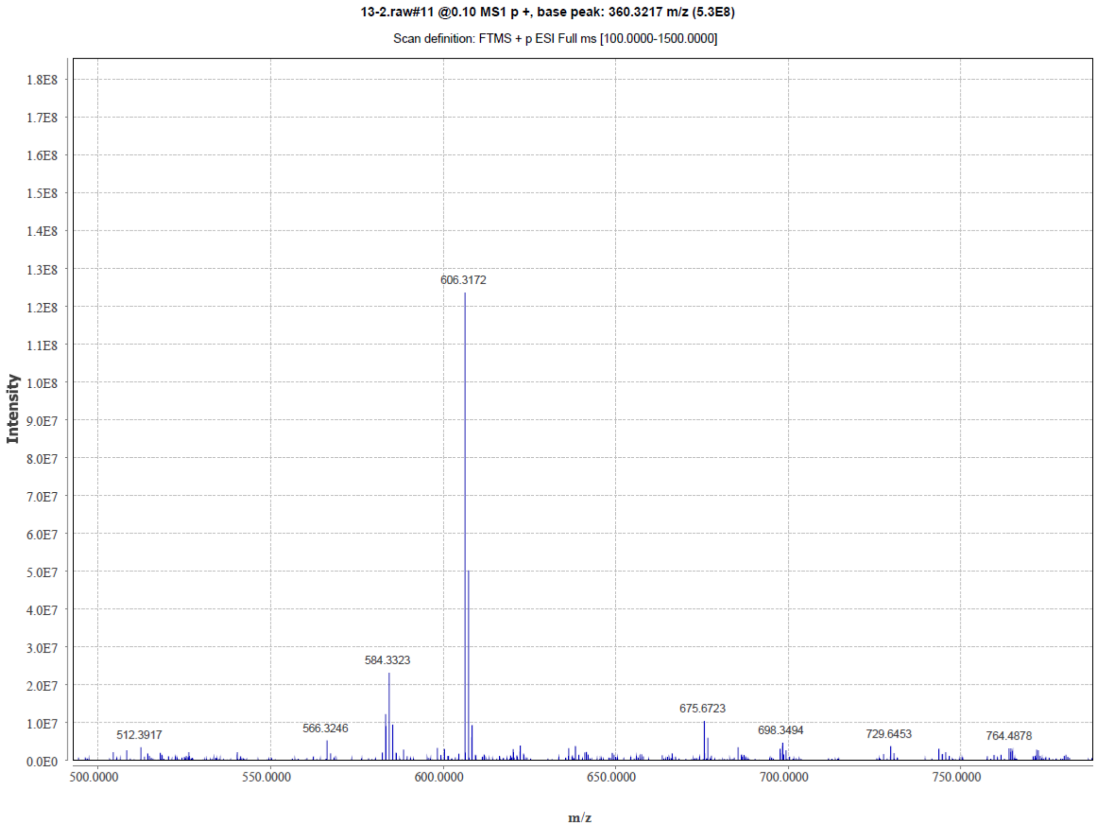

NMR and HRESIMS of compound 3

^{1}H-NMR spectrum of 3 in CD_3OD

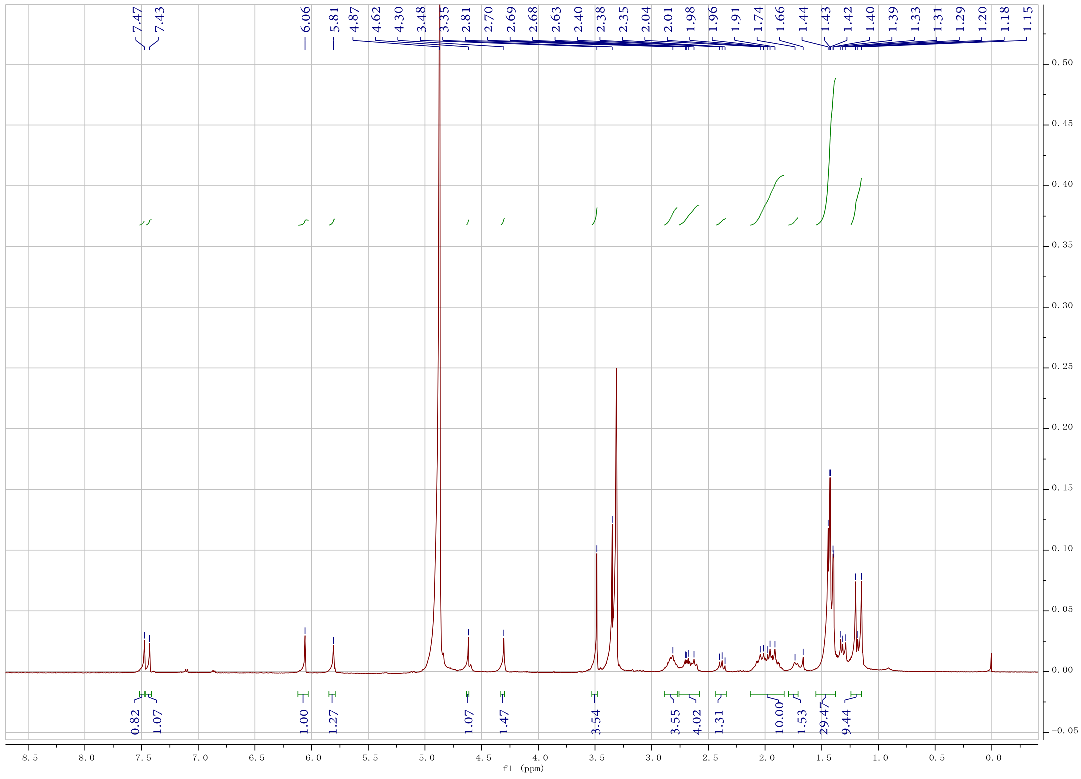

^{13}C-NMR spectrum of 3 in CD_3OD

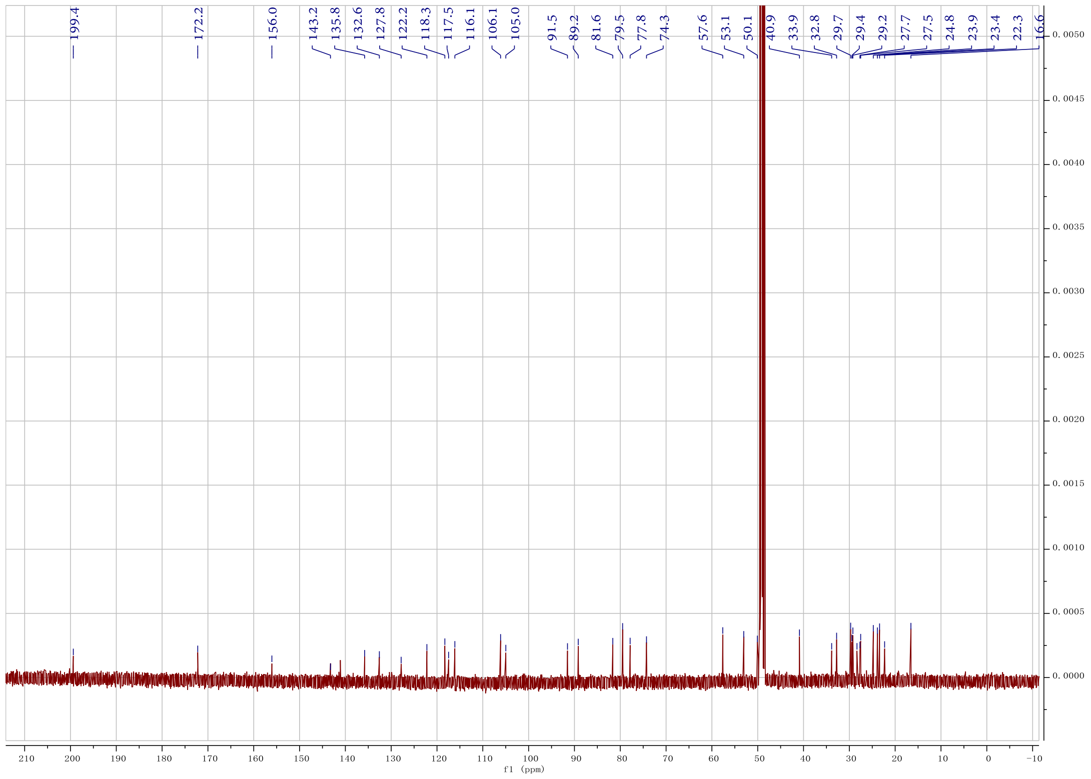

DEPT spectrum of 3 in CD_3OD

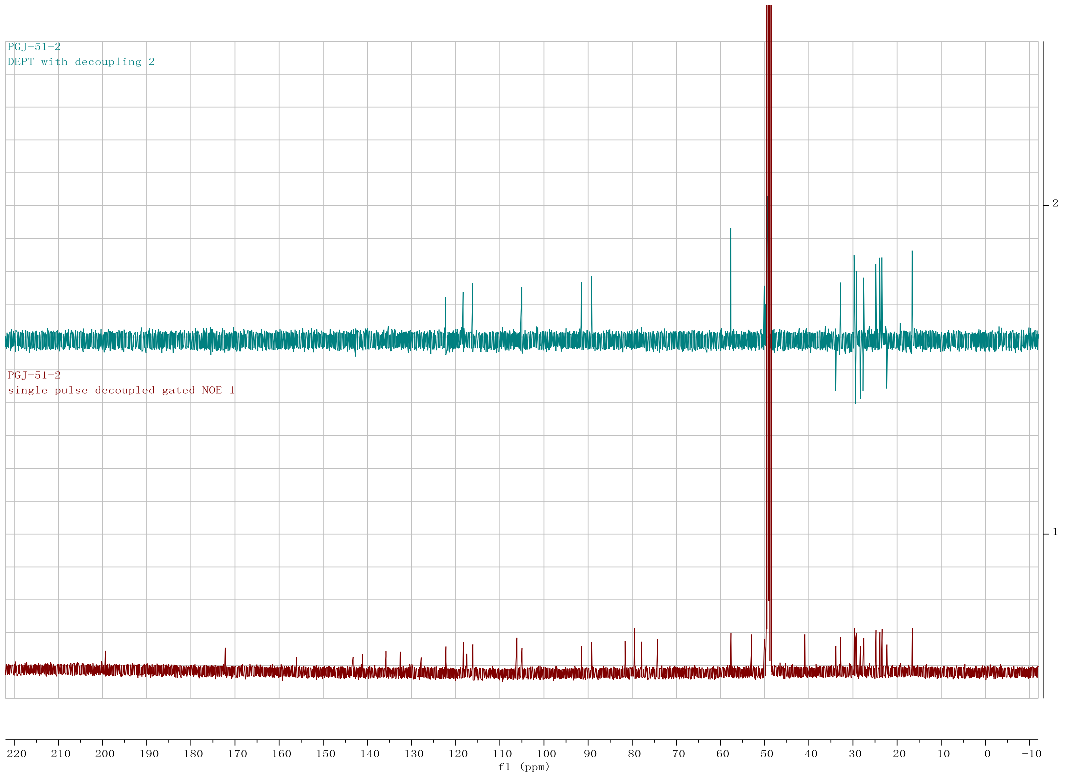

HSQC spectrum of 3 in CD_3OD

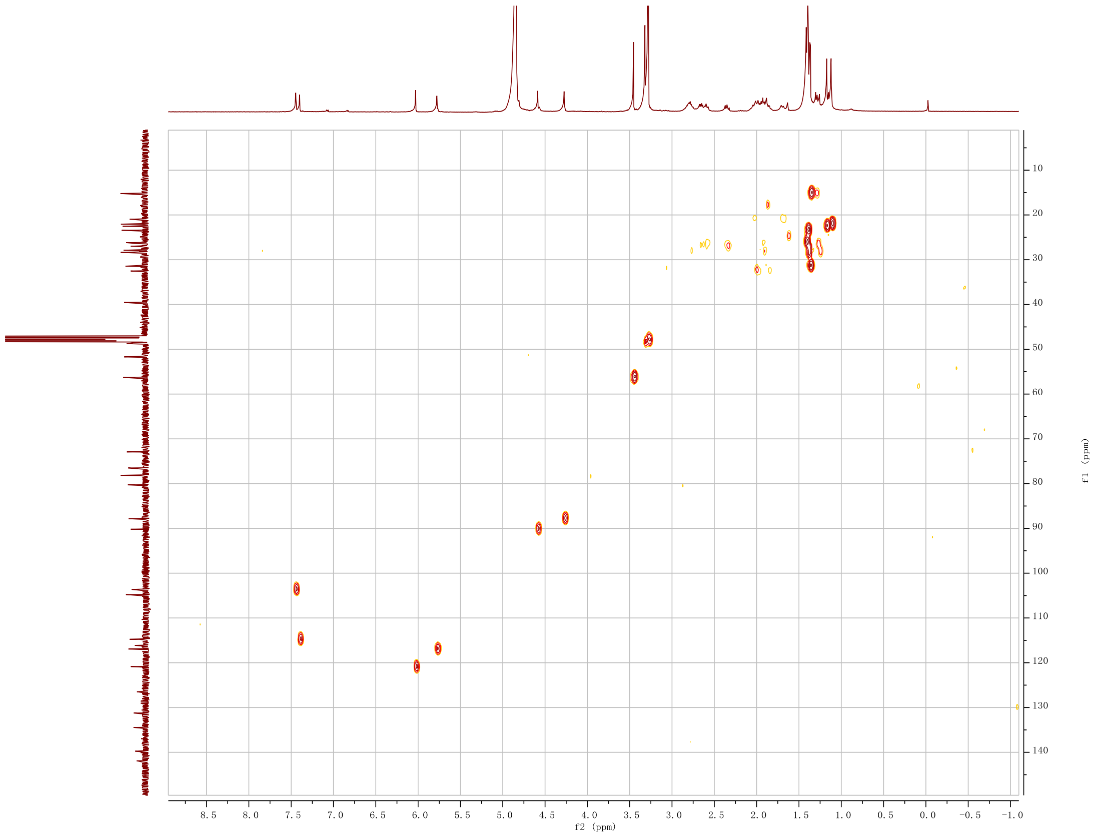

^1H-^1H COSY spectrum of 3 in CD_3OD

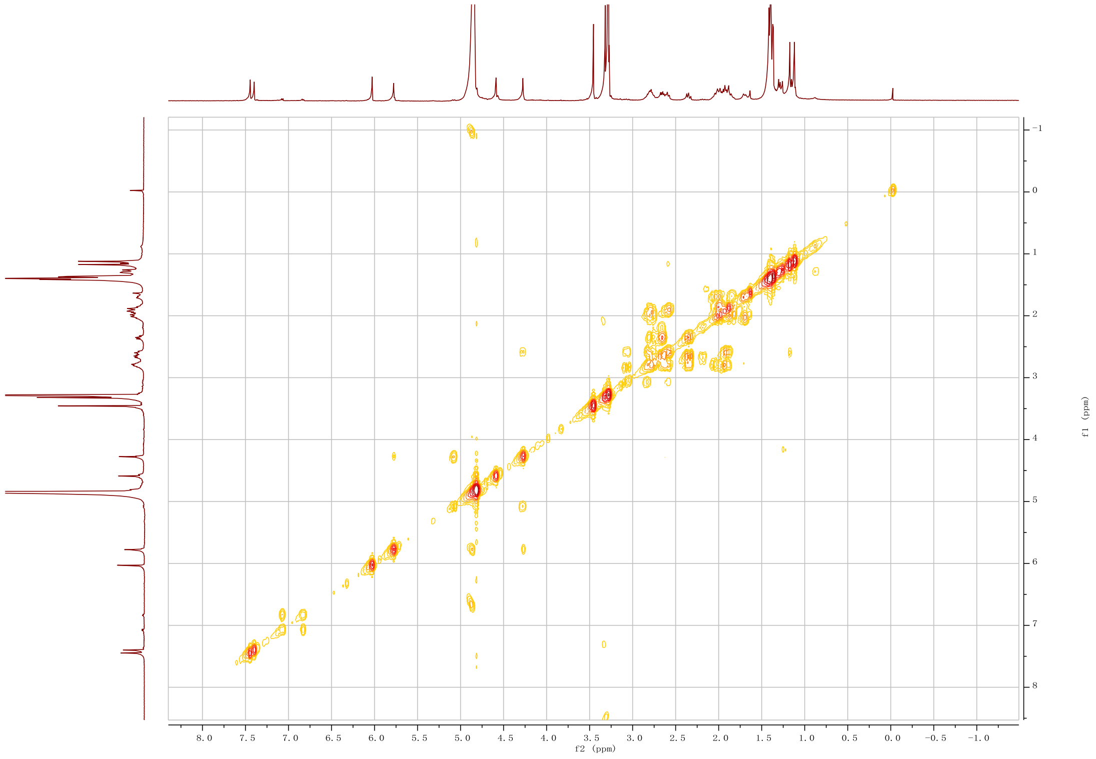

HMBC spectrum of 3 in CD_3OD

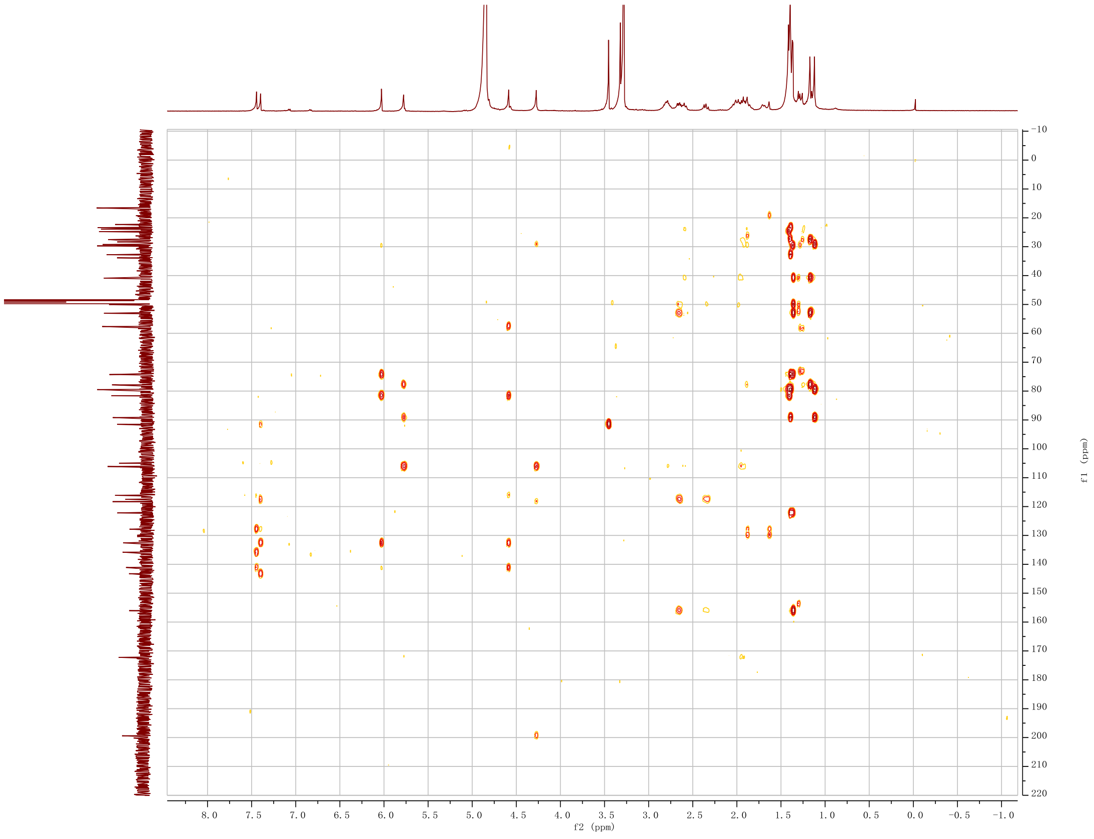

ROESY spectrum of 3 in CD_3OD

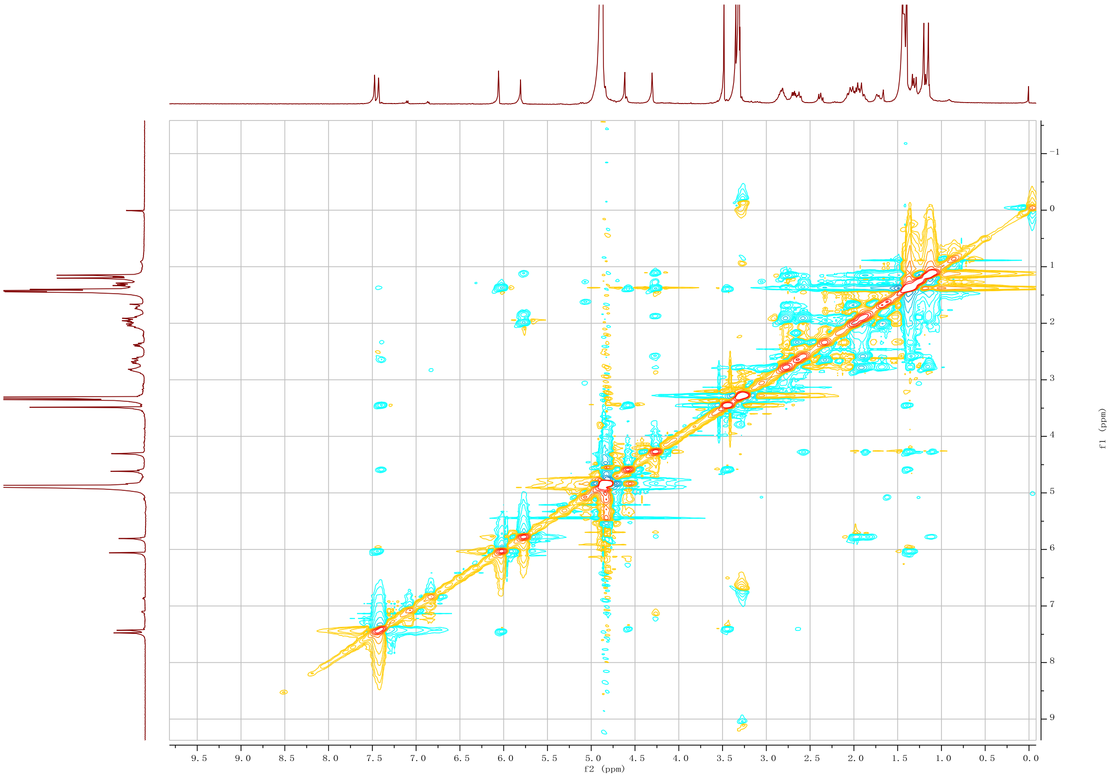

HSQC spectrum of 3 in DMSO-d_6

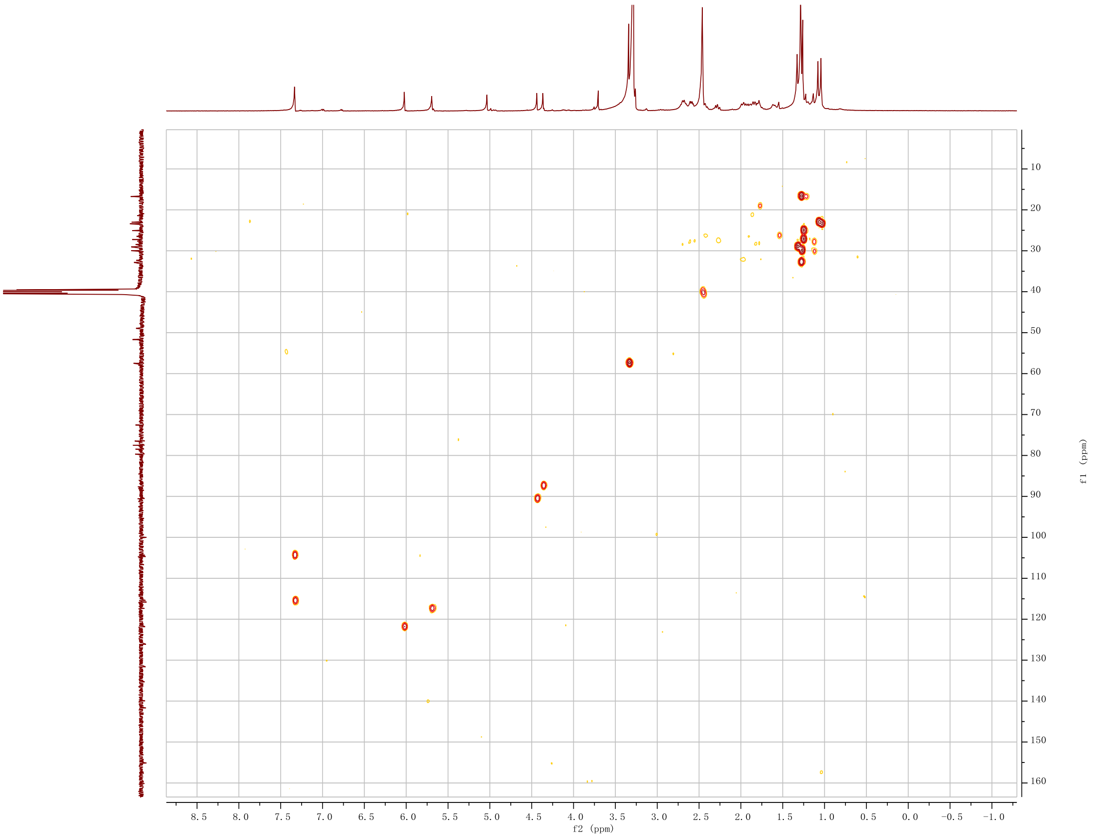

ROESY spectrum of 3 in DMSO-d_6

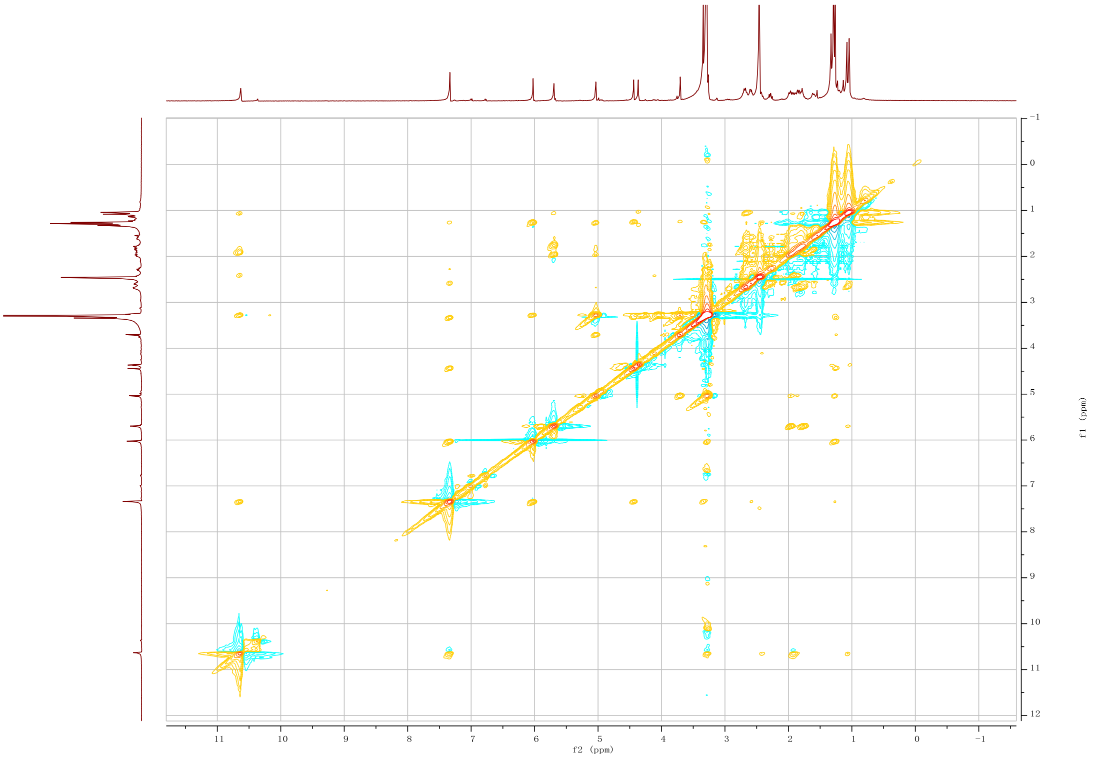

HRESIMS spectrum of 3

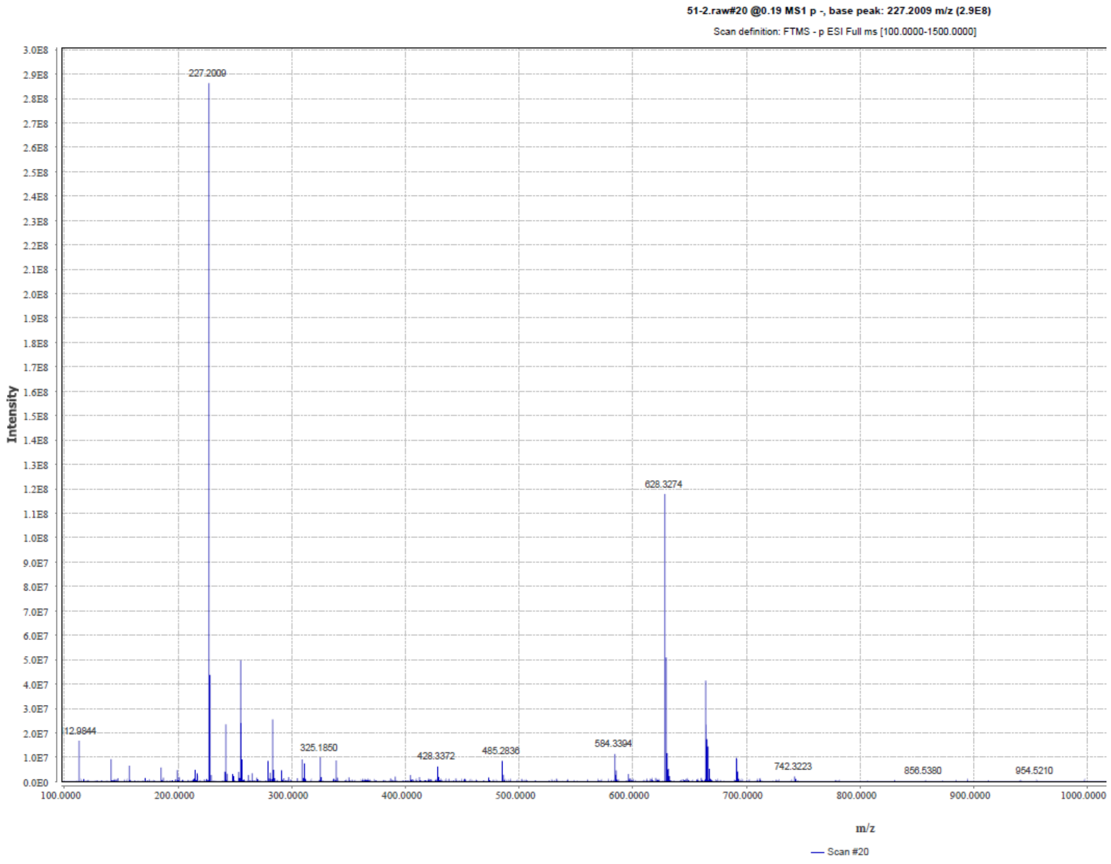

NMR and HRESIMS spectra of compound 4

^{1}H-NMR spectrum of 4 in CD_3OD

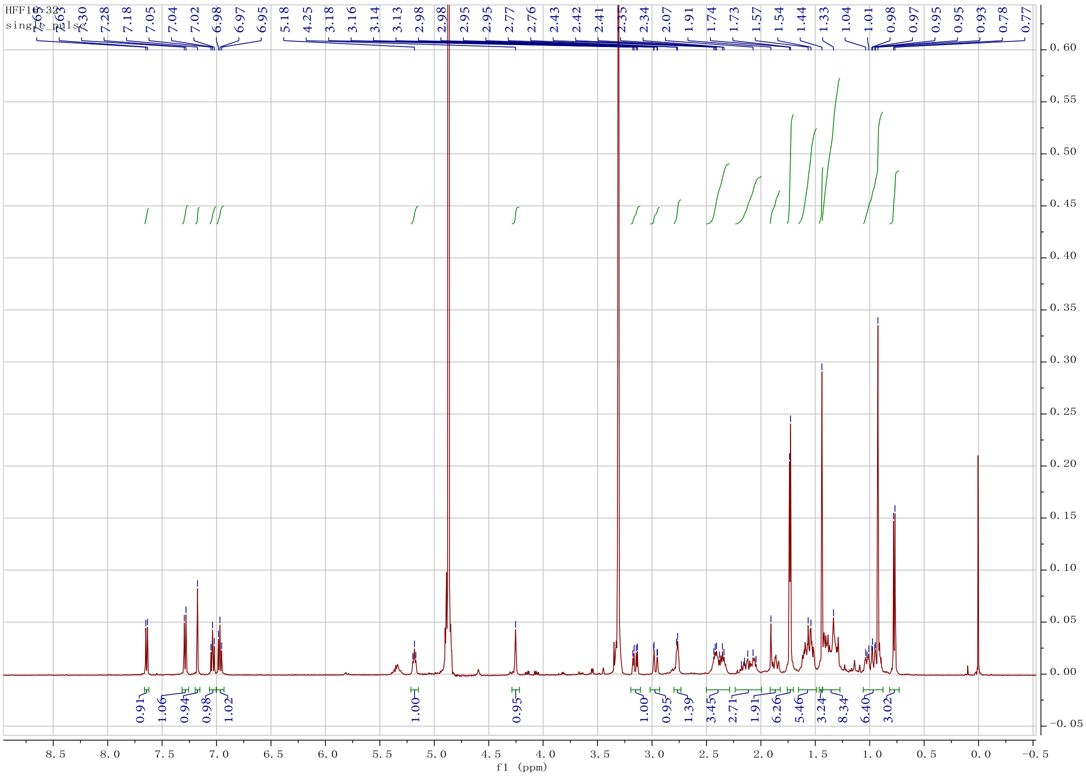

^{13}C-NMR spectrum of 4 in CD_3OD

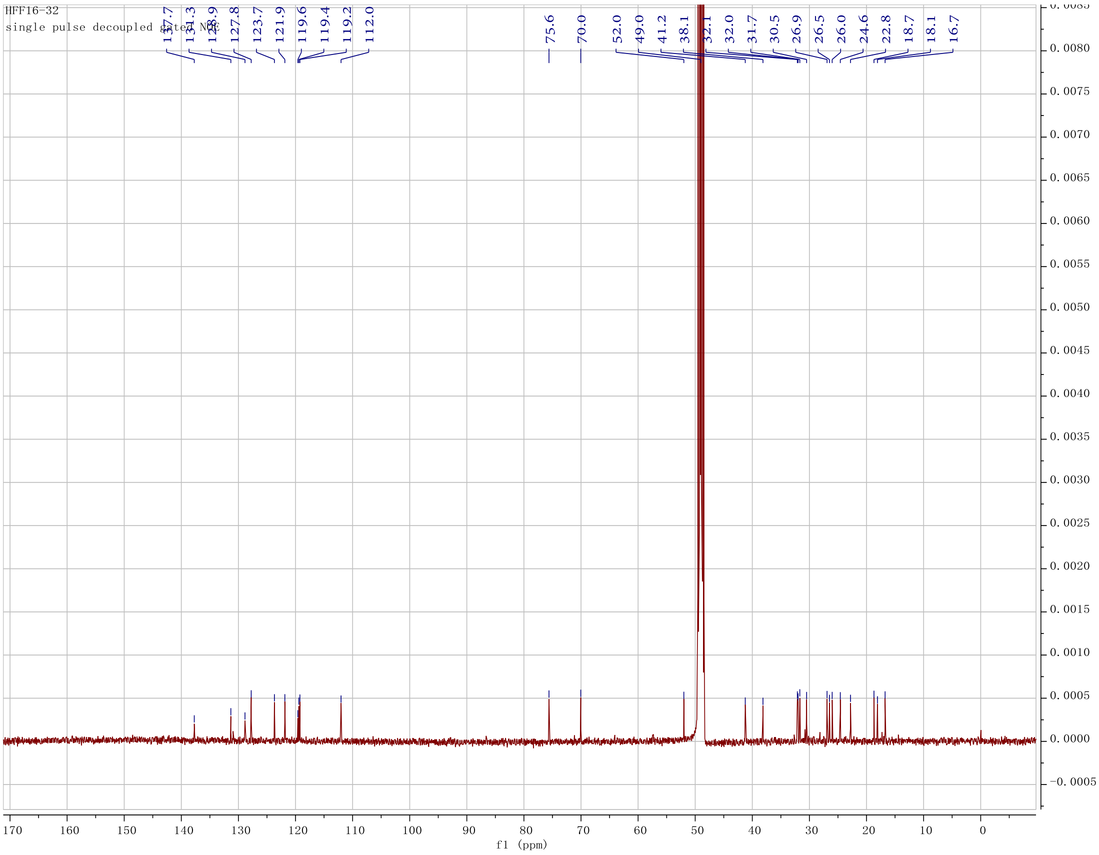

DEPT spectrum of 4 in CD_3OD

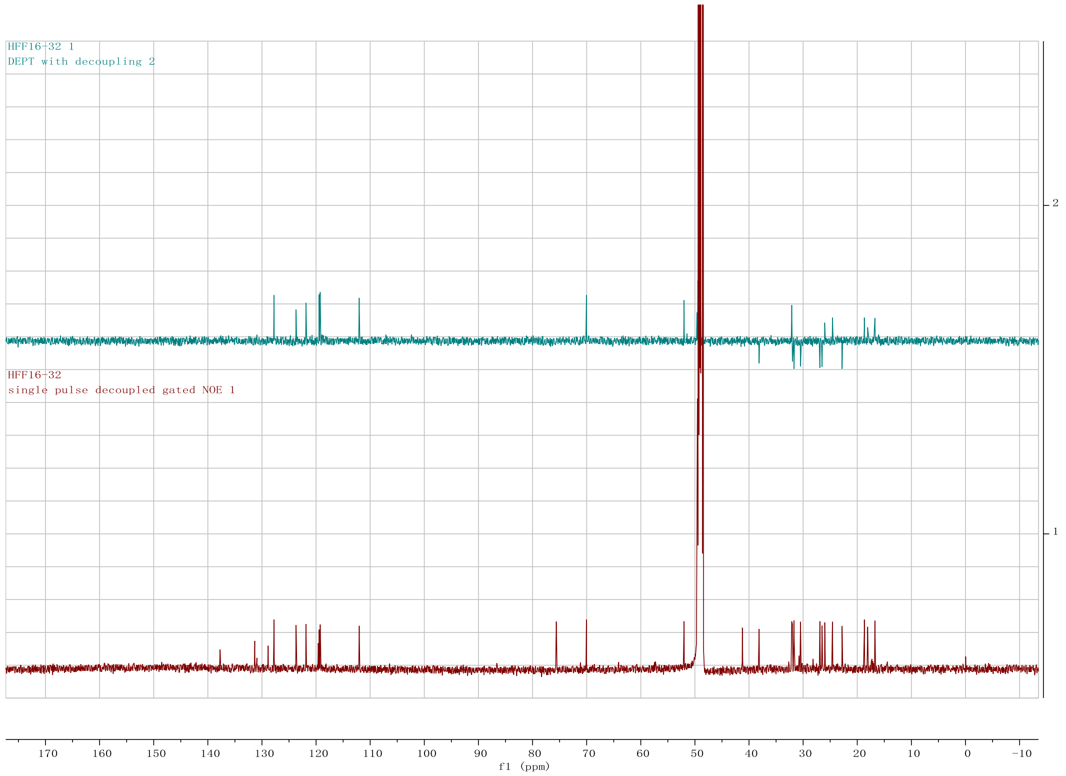

HSQC spectrum of 4 in CD_3OD

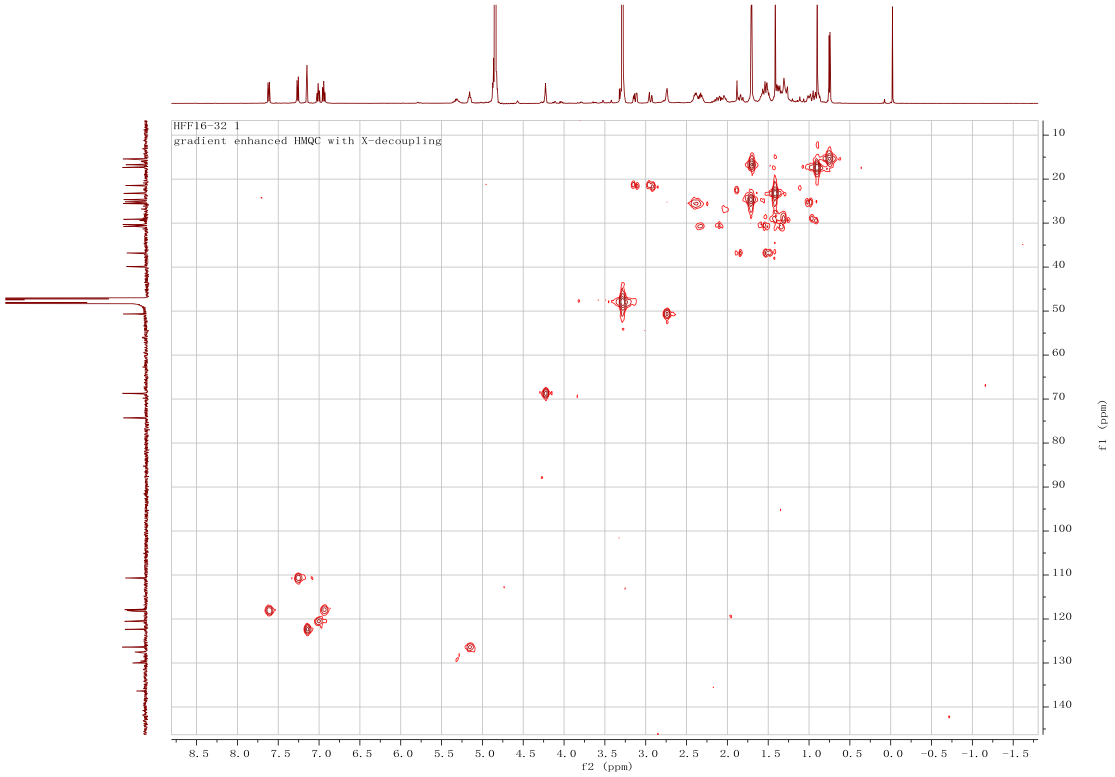

^{1}H-^{1}H COSY spectrum of 4 in CD_3OD

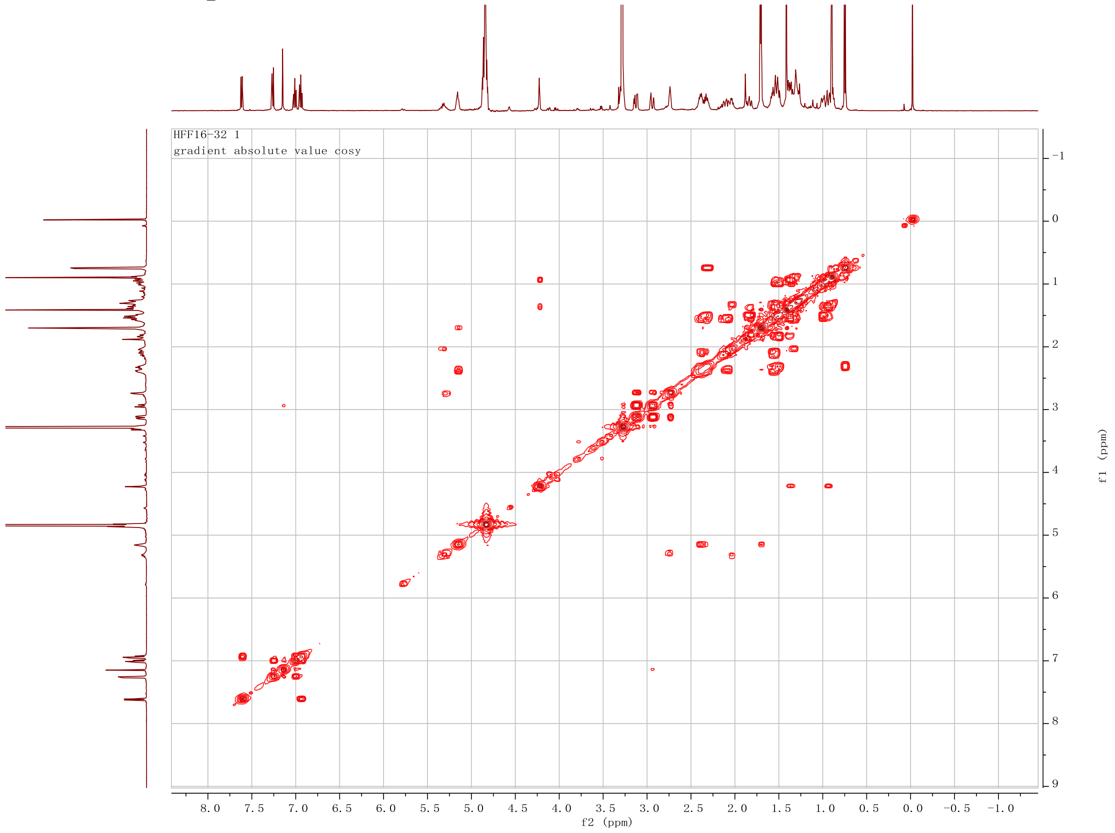

HMBC spectrum of 4 in CD_3OD

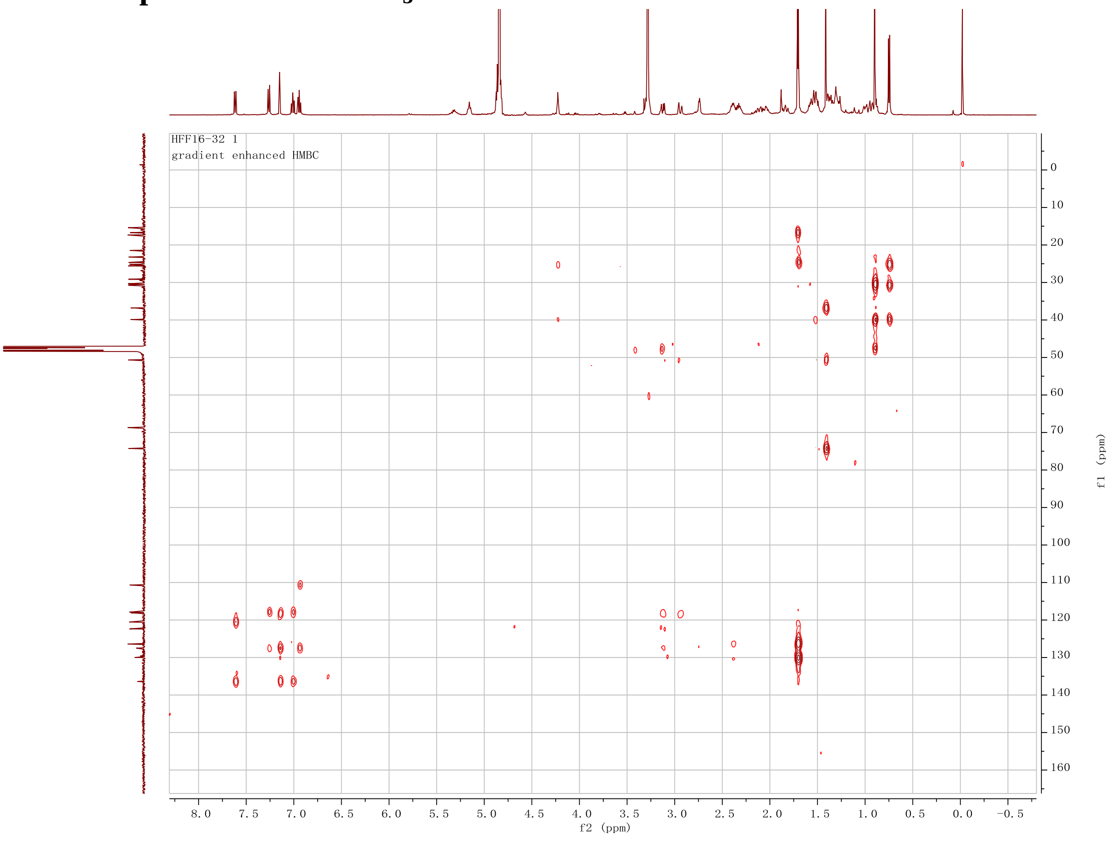

ROESY spectrum of 4 in CD_3OD

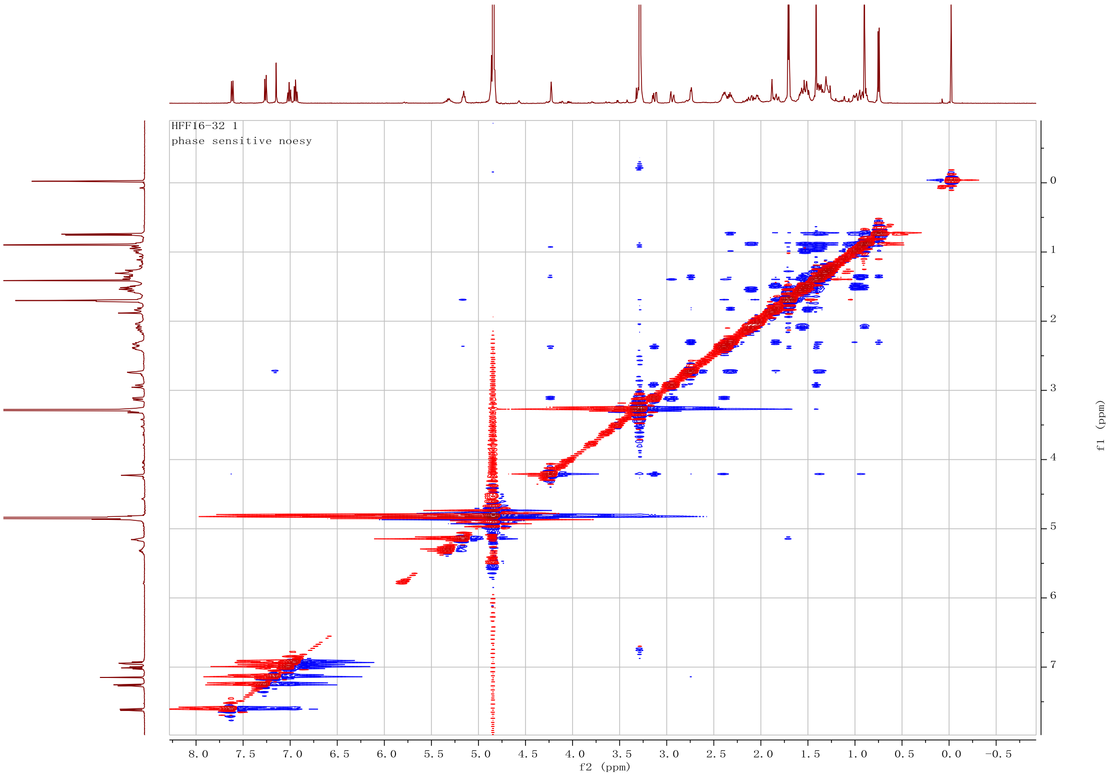

HRESIMS spectrum of 4

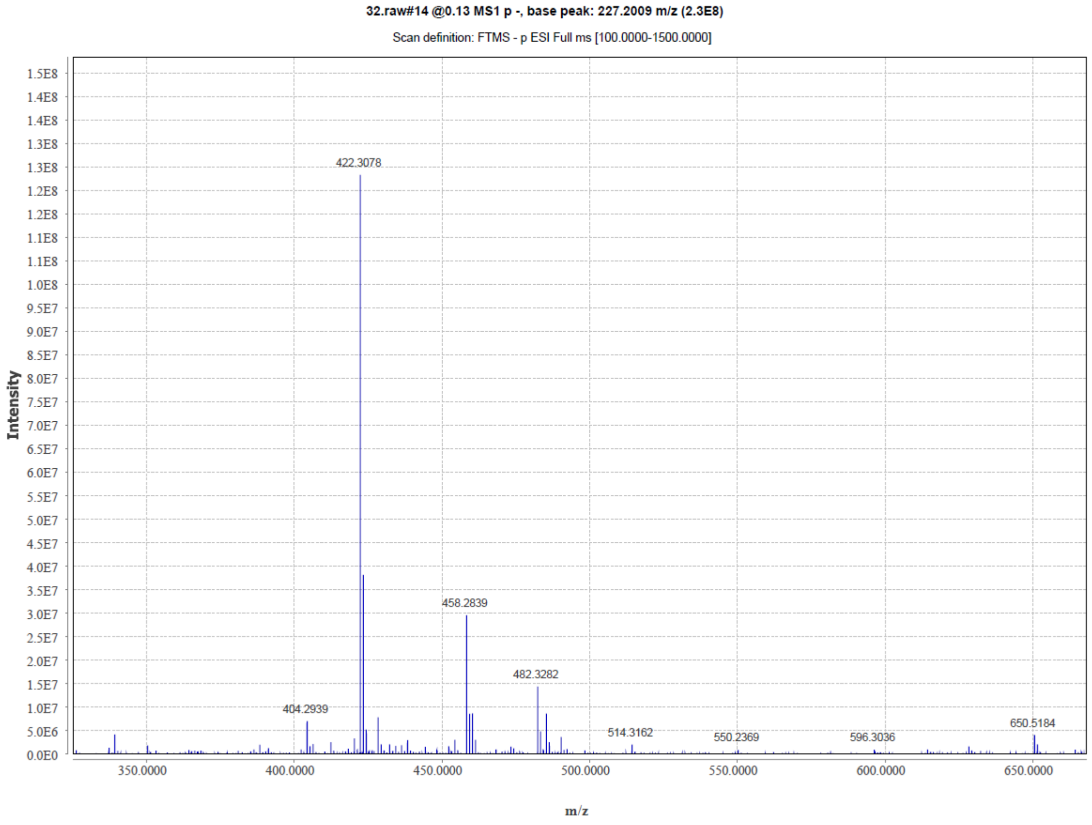

NMR and HRESIMS of compound 5

^{1}H-NMR spectrum of 5 in CD_3OD

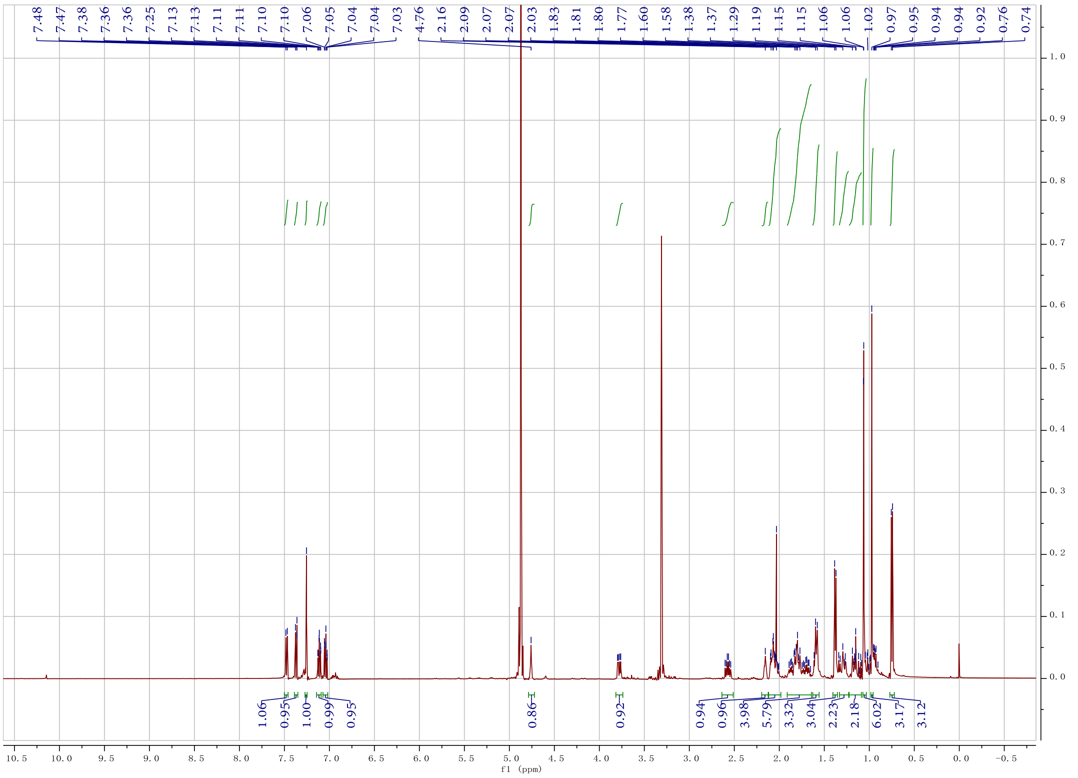

^{13}C-NMR spectrum of 5 in CD_3OD

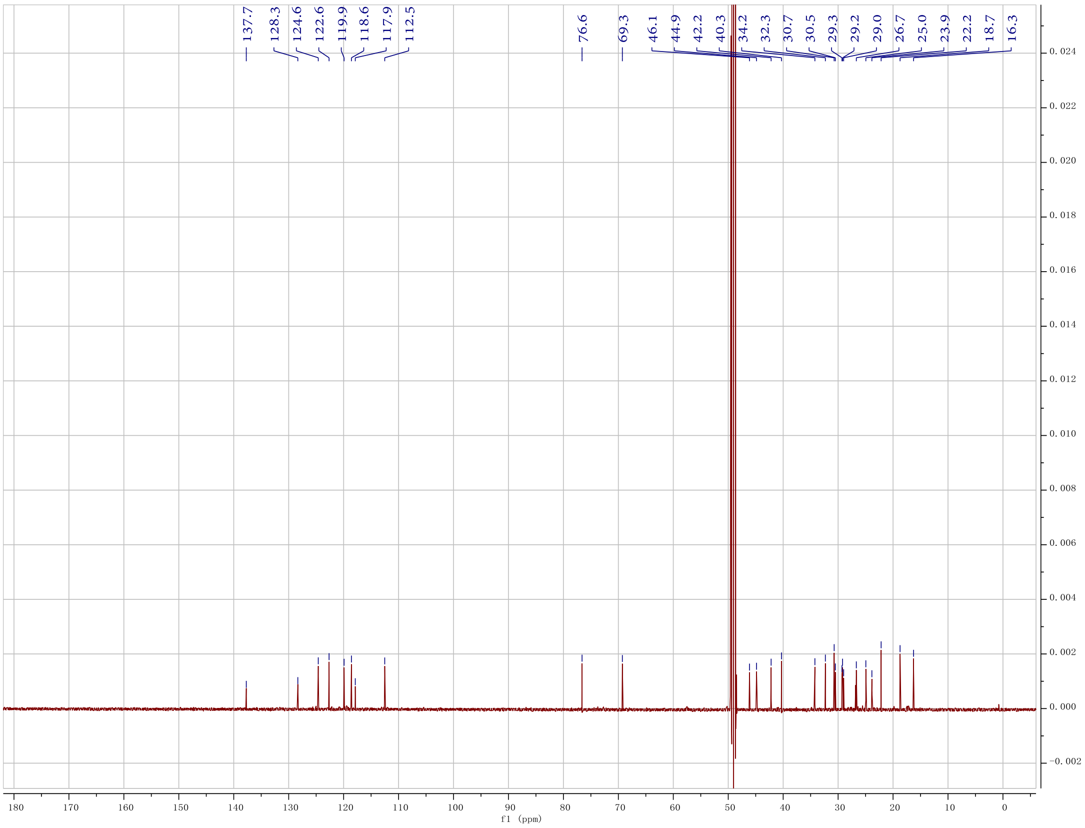

DEPT spectrum of 5 in CD_3OD

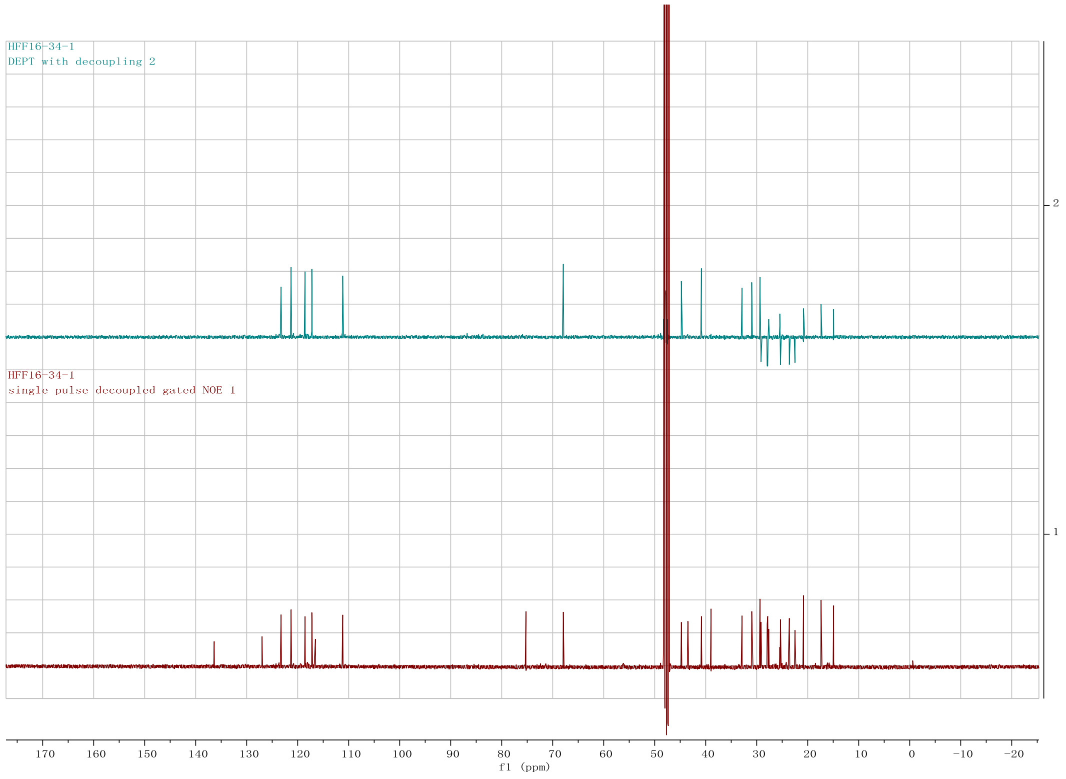

HSQC spectrum of 5 in CD_3OD

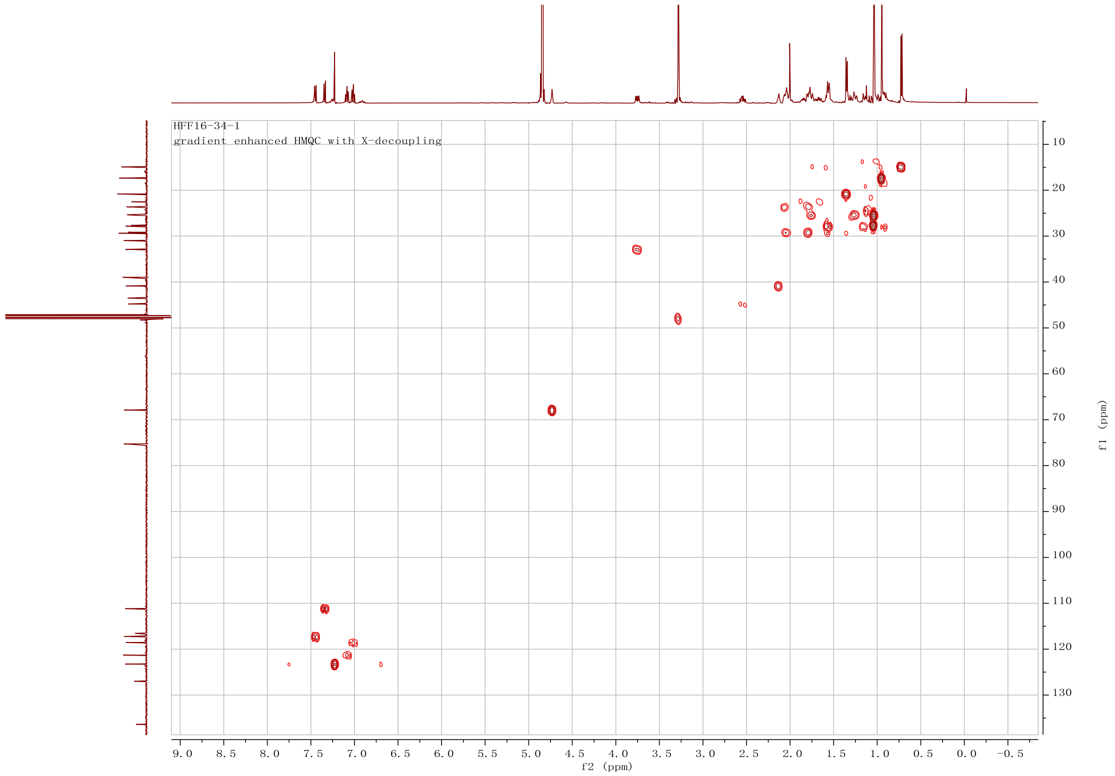

^{1}H-^{1}H COSY spectrum of 5 in CD_3OD

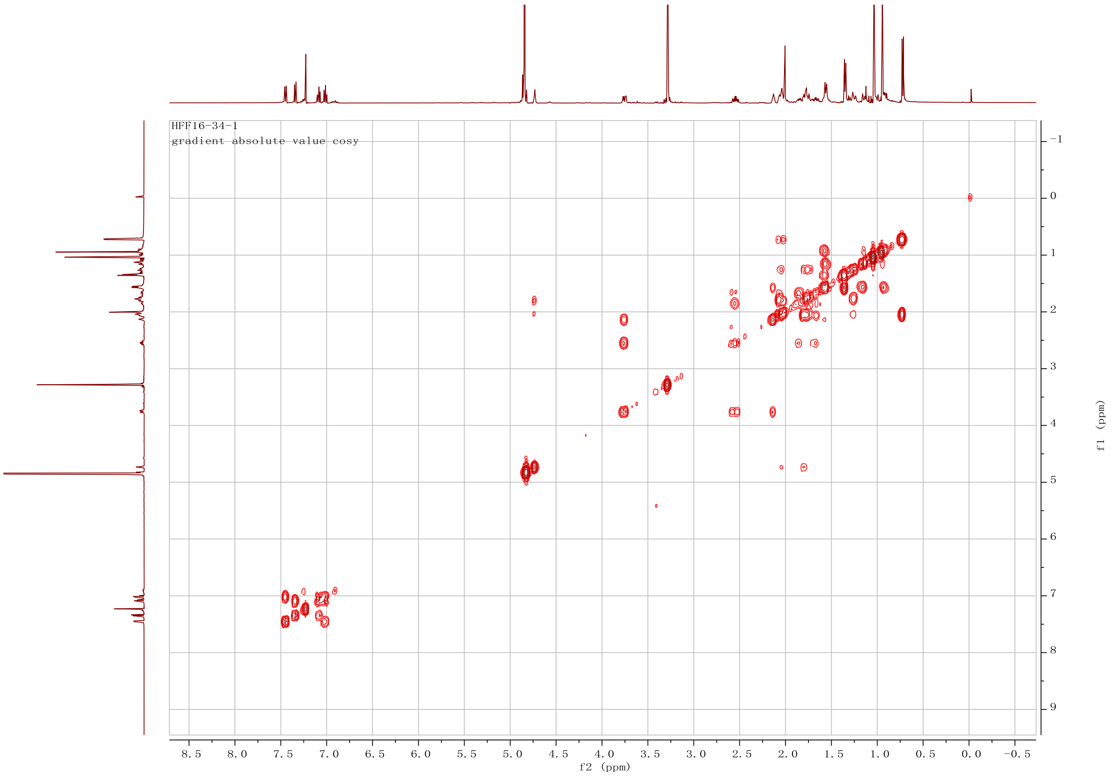

HMBC spectrum of 5 in CD_3OD

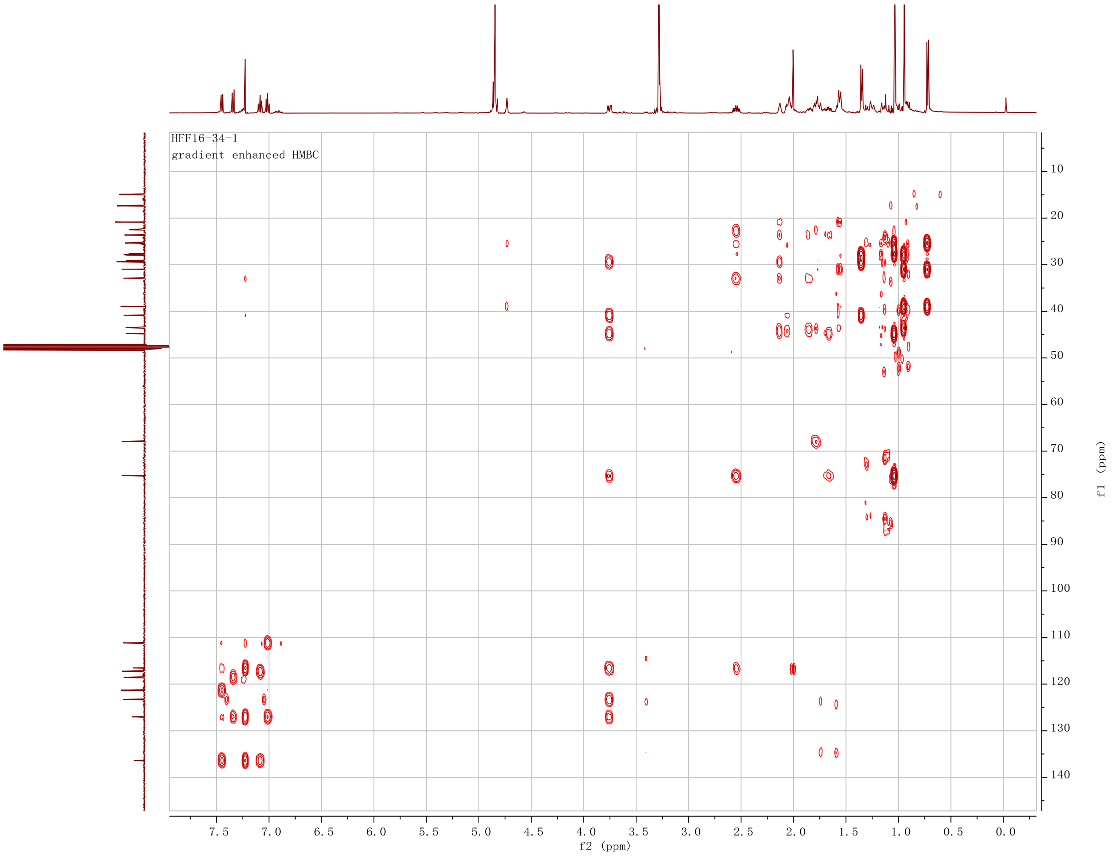

ROESY spectrum of 5 in CD_3OD

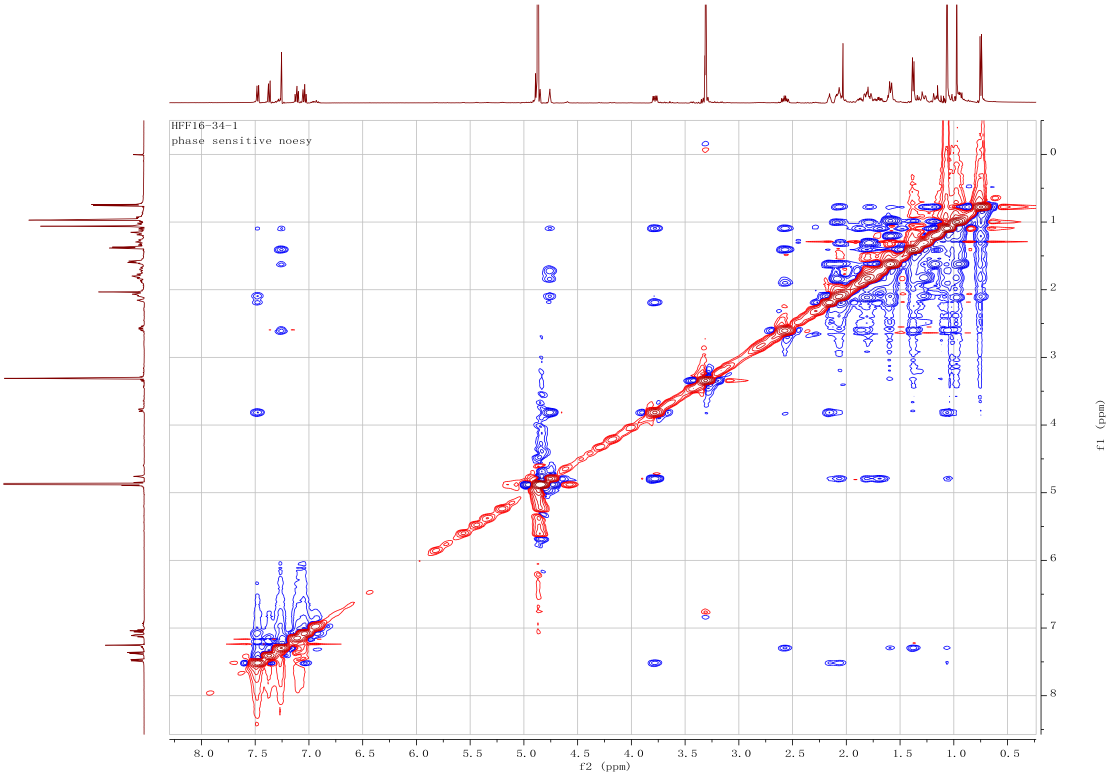

HRESIMS spectrum of 5

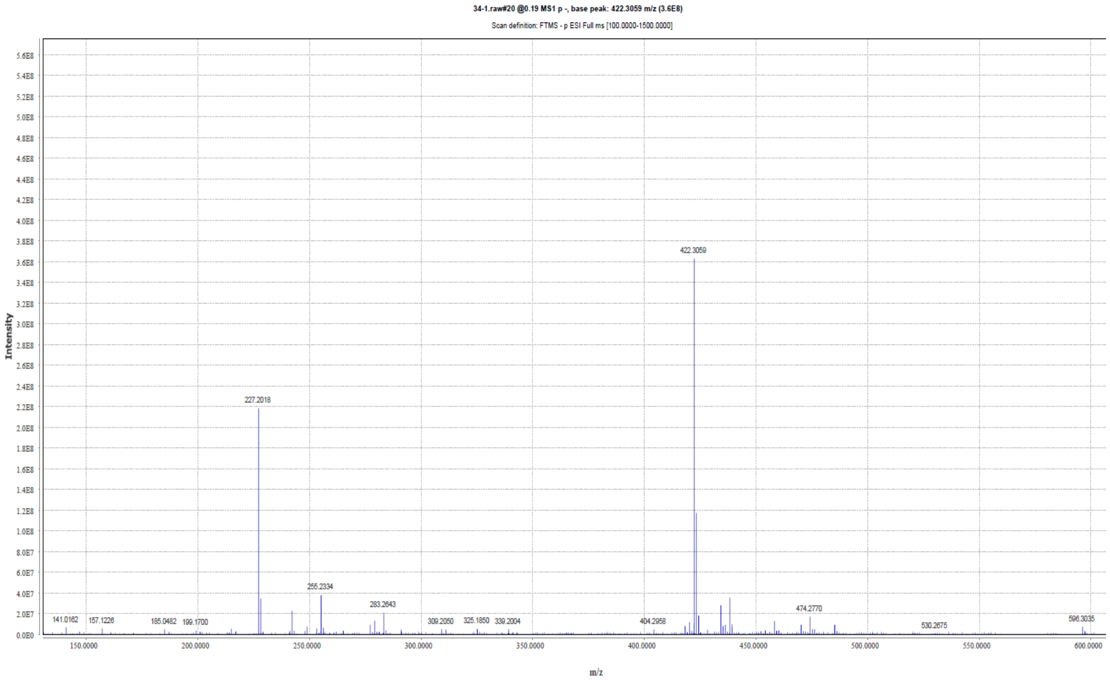

Supplement: Supplementary file 1 [file DataSheet1.PDF]
